# Novel application for graphene oxide-based ionanofluids in flat plate solar thermal collectors

**DOI:** 10.1038/s41598-024-67874-1

**Published:** 2024-07-30

**Authors:** I. Moulefera, A. R. Pastor, M. G. Fuster, J. J. Delgado-Marín, M. G. Montalbán, I. Rodríguez-Pastor, A. López-Pérez, I. Martin-Gullon, A. P. Ramallo-González, M. Alarcón, G. Víllora

**Affiliations:** 1 Chemical Engineering Department, Faculty of Chemistry, Regional Campus of International Excellence “Campus Mare Nostrum”, University of Murcia, 30071 Murcia, Spain; 2 Department of Chemical Engineering, Faculty of Science, University of Málaga, Andalucía TECH, 29071 Málaga, Spain; 3 Institute of Chemical Processes Engineering, University of Alicante, 03080 Alicante, Spain; 4 Department of Electrical Engineering and Electronic Technology, Universidad Politécnica de Cartagena, 30202 Cartagena, Spain; 5 Electromagnetism and Electronics Department, International Campus of Excellence in the European Context (CEIR) Campus Mare Nostrum, University of Murcia, Murcia, Spain

**Keywords:** Graphene oxide, Ionanofluid, Ionic liquid, [Emim]Ac, Flat plate solar thermal collector, Energy science and technology, Engineering, Materials science

## Abstract

This study presents new ionanofluids (INF) composed of 1-ethyl-3-methylimidazolium acetate ionic liquid (IL) and graphene oxide (GO) nanoparticles which have been assessed for the first time in an experimental flat plate solar thermal collector (FPSC). For this purpose, four types of INFs were synthesized, maintaining a constant concentration of GO nanoparticles dispersed in different base fluids: ionic liquid (IL/GO), a mixture of ionic liquid and water in varying concentrations (IL-water (75–25)%/GO and IL-water (50–50)%/GO), and water (Water/GO). These four INFs were characterized and their thermophysical and physicochemical properties were determined. The results indicated a 37.4% improvement in efficiency and up to a 2.5-fold increase in temperature within the collector when the IL was applied exclusively as the base fluid, compared to water. Furthermore, IL/GO demonstrated excellent stability, showing no signs of deterioration or nanoparticle precipitation two years after preparation and testing. These findings suggest that INFs based on IL and GO nanoparticles significantly enhance the efficiency of FPSC, presenting a promising option for solar energy applications and opening a new research avenue for INFs in the production of domestic hot water.

## Introduction

Climate change is defined as the shift in climate patterns mainly caused by an excess of greenhouse gas emissions. This excess disrupts the natural concentration levels, leading to an increase in heat trapped by the atmosphere, which is the main driving force behind global warming^1^. To mitigate this effect, renewable energies are presented as an alternative to fossil resources. Given the increasing energy needs in the current scenario and the rising energy prices, it is becoming increasingly essential to investigate and actively participate in the incorporation of new technologies.

Since sunlight is the most abundant and freely accessible energy resource on our planet, solar energy has emerged as the most significant renewable energy source to meet the excessive demand for energy, providing a promising solution to achieve sustainable development and combat global warming^2^. Solar energy is converted into electrical energy via photovoltaic (PV) modules. However, around 80% of the solar radiation absorbed by the PV panels is converted to heat or reflected, leading to an increase in the temperature of the panels, which negatively affects their efficiency in converting solar energy into electricity. Thus, the hybrid photovoltaic-thermal (PVT) solar collector stands as a more efficient option for enhancing thermal capacitance. Many researchers are currently assessing the possibility of evacuating the excess generated heat through a working fluid, such as water or air, and are exploring its potential for other purposes. The efficiency of the combined solar energy system is predominantly reliant on the thermo-optical properties of the working fluid and its performance under operating conditions. Conventional working fluids in solar energy technologies are water, ethylene glycol, the synthetic oil Therminol VP-1 or molten salts. Nevertheless, these working fluids possess inherent limitations. Challenges such as high vapor pressure, inadequate heat transfer properties, thermal instability at high temperatures, high melting points, and various other hindrances impede their practicality across diverse operating conditions. For instance, water is not feasible at elevated temperatures as it vaporizes above 100 ºC and exhibits an exceedingly high vapor pressure. Ethylene glycol and Therminol have suboptimal heat transport properties, while the molten salts have a high melting point^3^. Flat plate solar collectors (FPSC) typically operate below 100 °C during normal operation but can reach this temperature in severe weather conditions or failures. When working with water as a working fluid, overheating is a serious problem^4^, which must be mitigated using low vapor pressure fluids as working fluid. Therefore, the development of novel working fluids for medium-to-high-temperature applications is imperative.

Ionic liquids (ILs) exhibit several unique features that facilitate the development and synthesis of new heat transfer fluids by tailoring the cation–anion structure for desired physiochemical properties and specific target applications. Studies on the thermophysical properties of certain ILs have revealed that ILs have the advantages of high density and heat capacity, good thermal and chemical stability, and very low vapor pressure. These favourable thermophysical properties position ILs as promising candidates for their use as heat transfer fluids in medium- to high-temperature heat transfer systems^5^. Therefore, the application of ILs in solar energy technologies has been recently garnered attention, encompassing their use in solar collectors, solar troughs, solar photovoltaic-thermal panels and solar cells. ILs are now being considered as potential candidates for the next generation solar thermal fluids. Moreover, it has been demonstrated that the combination of nanomaterials and ILs exhibits tremendous potential as heat transfer fluids effectively enhancing their thermal properties^6^.

A nanofluid is a solid–liquid two-phase system that consists of a base fluid (such as water), in which particles with nanometric dimensions (1–100 nm), made of metals, metal oxides, or any solid particles with a higher thermal conductivity than water, are incorporated and maintained in suspension^7^. The nanofluid concept was first introduced by Choi and Eastman at the annual meeting of American Society of Mechanical Engineers in 1995^8^. Compared to solid materials, fluids exhibit significantly lower thermal conductivity. Thus, the use of fluids containing suspended solid particles appeared to be a promising solution for enhancing thermal conductivity. Nanotechnology has already demonstrated enormous success in the solar field, with thermal conductivity being influenced by several factors including temperature, size, shape, and concentration of the nanoparticles^9^. Nanofluids have emerged as a focal point of research for scientists worldwide due to their recognition as a new and advanced type of working fluid, due to their superior properties such as higher thermal conductivity and enhanced stability. Before incorporating nanofluids into a thermal system, the preparation of a stable colloidal suspension is essential. Physical methods such as stirring, sonication, homogenization, microwave treatment, plasma or laser ablation, as well as chemical methods, such as covalent or non-covalent functionalization can be employed for nanofluids preparation. In fact, a combination of them may prove to be the most effective approach (e.g., high shear stirring combined with sonication, or covalent functionalization alongside stirring). Nanofluid properties typically subjected to measurement include thermal conductivity, viscosity, density, specific heat capacity and electrical conductivity. The stability and properties of nanofluids are primarily influenced by (i) the type of nanoparticles (ii) the type of base fluid, and (iii) the chemical or physical processes used for nanofluid preparation^10^.

A new generation of nanofluids has recently emerged with the use of ILs as a base fluid, as first reported in the pioneering work of Nieto de Castro et al.^11^, who introduced the term ionanofluids (INFs). ILs demonstrate remarkably high stability behavior, yielding very stable suspensions with many nanomaterials, of different nature (polymeric, metallic and oxides). At first step, round-type nanomaterials such as alumina and silica yielded great enhancements of properties, due to the ease of processing and handling^6^. However, the most notable improvements in thermal conductivity were reported by Jóźwiak et al.^12^, who observed substantial increases up to 70% with high aspect ratio carbon nanomaterials, such as single and multiwall carbon nanotubes (SWCNT and MWCNT), especially with extra-long and straight CNT. On the other hand, these authors^12^ also indicated that rounded-shaped carbon nanomaterials such as fullerenes or carbon black did not produce any increase at all. Nevertheless, it should be noted that the results reported in the literature are very scattered, often heavily reliant on the specific properties of the nanomaterials. For instance, notable enhancements of approximately 30% in thermal conductivity were reported for graphene nanoplatelets (GNP)^13^, whereas nearly no increase was observed with either graphite flakes^12^ or nested MWCNT^11^. Consequently, there is a significant reliance on the dispersion techniques used in the preparation of nanofluids, as it is well known from the polymer nanocomposite field, and scarce efforts have been put into this aspect. Moreover, it is evident that favorable (although not always reproducible) results are obtained with carbon nanomaterials attributed to the high specific surface/energy density^14,15^ as well as exceptional solar light absorbing potential^16,17^ compared to other metal and nonmetallic nanoparticles. The incorporation of these advanced nanoparticles into the base fluid notably amplify optical and thermophysical properties. Therefore, considering the capacity of advanced nanoparticles to fine-tune the thermal and optical characteristics of ILs, recent research exploring the use of INFs for solar energy conversion have been strongly encouraged. This endorsement highlights their potential applications in solar energy systems designed to harness substantial quantities of radiation.

As previously mentioned, in recent years, numerous studies have been published on the use of INFs based on carbon nanoparticles for solar energy applications^3,18^. However, to the best of our knowledge, these INFs have not yet been applied either industrially or at the laboratory scale. These studies have focused, not only on the synthesis of innovative INFs, but also on simulations to demonstrate their potential future applications^19–23^. This work is significant as it represents the first instance of applying an ionanofluid with GO nanoparticles in a FPSC at the laboratory scale. The results pave the way for new opportunities in the industrial application of INFs, as it reveals and confirms the advantages of these compounds over water-based nanofluids, which have been the subject of various simulations^3,23^. The novelty of this work lies in its approach to addressing the research gap in improving the efficiency and stability of heat transfer fluids in flat-plate solar collectors (FPSCs). Traditional fluids often face limitations in thermal performance and long-term stability, whereas the developed INFs provide a viable alternative with superior properties. This study provides significant advancements in the field of renewable energy, specifically in enhancing the efficiency and lifespan of solar thermal systems. It also opens new avenues for further research and industrial applications, aiming to extend the use of INFs in various solar thermal energy systems beyond domestic hot water production. Current plans for scaling up tests on commercial solar devices will further elucidate the practicality and effectiveness of INFs, potentially transforming the landscape of solar thermal energy applications. In this study, the IL 1-ethyl-3-methylimidazolium acetate ([Emim] Ac) has been used. Four INFs composed of [Emim] Ac or [Emim] Ac/water mixtures as the base fluid and graphene oxide (GO) as nanoparticles were prepared. As comparison, a nanofluid with only water as base fluid and GO as nanoparticles has been also prepared. Their characterization was carried out by assessing their physicochemical and thermophysical properties. Finally, various tests were performed using an experimental solar collector to ascertain their temperature profiles and efficiency.

## Materials and methods

### Materials

The IL 1-ethyl-3-methylimidazolium acetate (≥ 95% purity), [Emim] Ac, was supplied by IoLiTec (Heilbronn, Germany) and used as received. The water used to achieve the IL-water mixtures was obtained from a Merck Millipore System Milli-Q with a resistivity value of 18.2 MΩ·cm. Natural expanded graphite was purchased from Imerys (Bodio, Switzerland). KMnO_4_, NaNO_3_, H_2_SO_4_ (95%) and HCl (37%) were supplied by VWR and H_2_O_2_ (33 vol%) was supplied by Fisher. Tables 1 and 2 show the specifications of the used materials.

**Table 1:** Specifications of natural expanded graphite.

Purity	
Ash (810 °C)	0.15%
Moisture	0.10%
Density	
Scott (non bagged)	0.043 g/cm3
Surface area	
BET (single point)	22.5 sqm/g
Particle size	
Laser diffraction	
d10	10.0 µm
d50	37.8 µm
d90	79.1 µm
XPS	
C/O	20.8

**Table 2:** Specifications of ionic liquid 1-ethyl-3-methylimidazolium acetate.

Purity	≥ 95%
Water content	< 1%
Appearance	Yellow to red liquid

### Preparation of graphene oxide (GO)

GO was prepared using the Hummers-Offeman procedure with minor modifications. First, 1 g of graphite was mixed with 70 mL of H_2_SO_4_ and 1 g of NaNO_3_ and the mixture was stirred for 3 h at room temperature. Subsequently, 4 g of KMnO_4_ were slowly added, and the resulting mixture was stirred for 2 h. Then, the temperature was increased to 55 °C. After 1 h, the reaction was cooled to room temperature and poured into 90 mL of cold water with 70 g of ice stirring the mixture. After 1 min, 8 mL of concentrated H_2_O_2_ was added to stop the oxidation by reacting all manganese moieties to Mn^2+^ and to prevent MnO_2_ precipitation. The solid was filtrated and further washed with 160 mL of water for 30 min, adding 30 mL of HCl (20 vol%) to promote coagulation. The mixture was filtrated again. Finally, the obtained GO was dried 24 h at 60 °C.

### Preparation of ionanofluids

Five different suspensions with mass fraction of 1% (wt/wt %) of GO were prepared (see Table 3). Briefly, GO was initially dispersed in the fluid base (pure IL, IL/water mixture or pure water) under magnetic stirring for 10 min. Then, the sample was bath sonicated for 30 min at 25 °C and, finally, was probe sonicated with a Branson 450D high power ultrasonicator at 40% of amplitude with pulses of 59 s ON/29 s OFF for at least 4h. Due to the high temperature reached during ultrasonication, the experiments were carried out in an ice bath.

**Table 3:** Base fluid composition of the samples.

Suspension	IL content (wt/wt%)	Water content (wt/wt %)
IL pure	100	0
IL/GO	100	0
IL -water (75–25) %/GO	75	25
IL-water (50–50) %/GO	50	50
Water/GO	0	100

### Graphene oxide characterization

X-ray photoelectron spectroscopy (XPS) was carried out with a K-alpha spectrometer (Thermo-Scientific); the surface atomic O/C ratio was calculated by the integration of survey spectra, and analysis of functional groups bonded to C was performed by deconvoluting the C1s spectra. Thermogravimetric analysis (TG) was performed using a Mettler Toledo apparatus (TGA/SDT851e/LF/ 1600) in order to measure the weight loss. These experiments were carried out under He atmosphere from room temperature to 1000 °C at rate 10 °C/min. GO was explored by Transmission electron microscopy (TEM, JEOL model JEM-1400 Plus); isopropanol was used as the solvent for exfoliation of GO at a concentration of 0.1 mg/mL using a an ultrasonic tip (30W, 2 h with ON–OFF intervals 60–30 s); a drop of the suspension was deposited on a carbon-coated copper grid, evaporating the solvent at room temperature. Same GO suspension was deposited in another grid for exploration in high angle annular dark field scanning transmission electron microscopy (HAADF-STEM model FEI Talos F200X) equipped with Super-X EDS analytical system for high-speed elemental mapping with nanometer-scale spatial resolution. Infrared spectroscopy with attenuated total reflectance (IR-ATR) was performed with a BRUKER IFS 66 infrared instrument, using an ATR attachment.

Diffractograms of the GO were recorded on a Bruker diffractometer (D8-Advance model, Ettlingen, Germany) equipped with a KRISTALLOFLEX K 760-80F X-ray generator (Power = 3000 W, Voltage = 20–60 kV and Intensity = 5–80 mA) which has an X-ray tube with copper anode (λ = 1.54056 Å). The equipment was operated at 40 kV and 40 mA with 2θ varying from 4 to 50° with a step size of 0.05°.

### Ionanofluid characterization

Once the INFs were obtained, their size distribution was analysed using Dynamic Light Scattering (DLS) with a Malvern Zetasizer Nano ZSP instrument from Malvern Instruments Ltd. (Grovewood, UK). The instrument was equipped with a laser emitting at 4 mW power and at wavelength of 633 nm. This analysis determined the hydrodynamic intensity-weighted averaged diameter, known as Z-average.

The suspension stability was analysed using the Turbiscan^®^LAB™ (Formulaction Inc, Toulouse, France) equipment by the Multiple Light Scattering (MLS) technique. In operation, the sample dispersion is housed within a cylindrical glass cell and left there for 48 h. Illumination is provided by an electroluminescent diode emitting near-infrared light at a wavelength of 880 nm (λ_air_ = 880 nm). The Turbiscan® LAB™ employs two synchronized optical sensors: one for capturing light transmitted (T) through the sample at a 180° from the incident light (transmission sensor), and the other for detecting light that is backscattered (BS) by the sample at a 45° from the incident radiation (backscattering detector)^24^. The dimensionless Turbidity Stability Index (TSI) was calculated using Eq. (1) for all the INFs^24^:

1 $$TSI = \mathop \sum \nolimits_{{t_{i} }} ^{{\mathop \sum \nolimits_{{h_{i} }} \left| {scan_{{t_{i} }} \left( {h_{i} } \right) - scan_{{t_{{i - 1}} }} ~\left( {h_{i} } \right)} \right|}} /H$$

where H is the total sample height and scan_i−1_(h_i_) and scan_i_ (h_i_) are the intensity of scanning light (BS if T < 0.2%, T otherwise) at height h_i_, respectively, at time t_i−1_ and t_i_.

Thermal conductivities were determined using a TEMPOS thermal properties analyzer (Meter, WA, USA) employing the technique called hot wire at the temperature range of 20–60 °C. Each sample was measured at least three times at every temperature with a precision of ± 0.0075 W·m^−1^·K^−1^. The measurements were carried out by placing the samples into a thermostatic bath at the desired temperature, ensuring that no movement was induced by using a clamp. The samples were maintained at a stable temperature until equilibrium was achieved. Subsequently, the water flow was reduced to 10% of the bath capacity to ensure that the measurements were not affected by the movement. This procedure was repeated continuously overnight, with a series of measurements taken at 30 min intervals.

Viscosities were measured using a Bohlin Visco 88 rotational viscometer (Malvern, Worcestershire, UK) with a precision of ± 0.0025 Pa s at the temperature range of 20–70 °C controlled by a thermostatic bath. The measurements were performed at least in triplicate.

Specific heat capacity measurements were performed by Differential Scanning Calorimetry (DSC 822E, Mettler-Toledo, Ohio, USA) using an IsoStep method with sapphire as the comparison standard. Nitrogen was used to purge the system with a flow of 50 mL·min^−1^. All measurements were taken at temperatures ranging from 20 to 70 °C. The IsoStep method consists of an alternating series of isothermal steps of 2 min followed by non-isothermal steps of 2 min with heating rate of 2.5 °C/min. The measurements were taken in triplicate.

Thermal stabilities of INFs were analyzed using a TGA/DSC 1HT thermogravimetric analyzer (Mettler-Toledo, Barcelona, Spain). The samples were heated from 30 to 800 °C at a rate of 10 °C/min under a nitrogen atmosphere.

Densities were measured using an oscillating U-tube DMA 4500 M densitometer (Anton-Paar, Graz, Austria) at the temperature range of 20–70 °C. The temperature was controlled by a Peltier system. Standard uncertainty of density is 0.05 kg.m^−3^. The measurements were done at least three times.

The refractive index was measured by a RX 5000α refractometer (ATAGO CO., Tokyo, Japan) at 589 nm sodium wavelength and temperatures from 20 to 60 °C. The standard uncertainty of refractive index is 0.0002. The measurements were carried out in triplicate at least.

### Solar collector tests

To assess the behavior of the INFs, several tests were carried out in the solar thermal pilot plant described below. This plant had been previously been used to test water and a water-aluminium oxide (alumina) nanofluid, resulting in a significant improvement in the thermal efficiency of the collector^25^. IL/GO INF was chosen for experimentation in the solar plant, and to examine and compare the effect of the base fluid on the efficiency of the collector, water and IL were also investigated.

**Figure 1:**
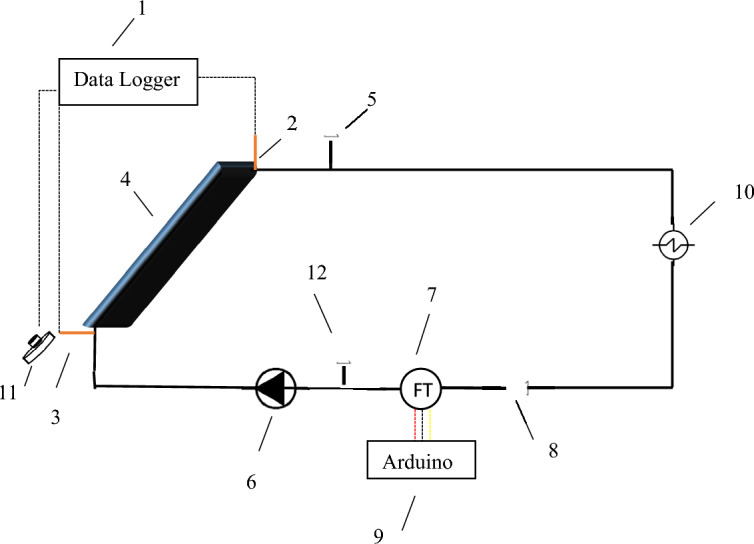
Simplified schematic of the solar installation. (1) Data logger system. (2,3) Pt100. (4) Flat solar collector. (5) Air purge. (6) Centrifugal pump. (7) Flowmeter. (8) Flow control valve. (9) Microcontroller. (10) Heat exchanger. (11) Pyranometer. (12).

The solar thermal pilot plant (Fig. 1) is based on a flat plate solar collector (FPSC) comprising small solar thermal collectors. The collector consists of a flat aluminum plate connected to copper tubing, a methacrylate cover, all inserted into a carefully insulated support box. Table 4 lists its main elements and dimensions.

**Table 4:** Characteristics of the pilot solar collector.

Construction type	Sheet and parallel tubes
External dimensions (mm^3^)	556 × 348 × 108
Aperture dimensions (mm^2^)	534 × 330
Aperture area (m^2^)	0,176
Flat plate material	Al
Flat plate thickness (mm)	1,5
Number of risers	3
Tube (risers and buffers) diameter (mm)	12
Tube thickness (mm)	1
Transparent cover	Glass
Insulation	Mineral wool
Insulation thickness (mm)	27
Collector box	Wood

The experimental setup was filled with either water, IL, or INF. The prepared INF underwent to separate stationary performance tests according to UNE-EN 12,975^26^. The collector was mounted on a mobile platform that facilitated adjustments in its orientation according to the solar position. The coplanar arrangement of the pyranometer ensured the correct measurement of the incident radiation. The tests were carried out with the collector positioned at 45° with respect to the horizontal plane. The plants silicone tubes enabled the confirmation of the absence of bubbles in the hydraulic circuit. The tests were conducted on clear sunny days of the months of June and July 2022.

The pilot plant is equipped with a pump capable of a maximum flow rate of 800 L/h, an air-cooling coil, silicone tubes, and ventilation control valves. The plant is also outfitted with flow meters, pyranometers, and a set of temperature probes (RTDs and T-type thermocouples) to monitor the environmental and operational factors required for conducting solar tests. The most crucial variables are continuously gathered by the data logger. The measuring devices used to evaluate the solar system are summarized in Table 5.

**Table 5:** Measurement instrumentation of the pilot experimental solar plant.

Data logger	Keysight 34972A Data logger with 34901A multiplexer
Fluid and ambient temperatures	4W RTD (locally calibrated)
Flat plate and tube temperatures	Type T thermocouples (locally calibrated)
Pyranometer	LP-PYRA 03 (2nd Class) of Delta Ohm
Flowmeter	Miniturbine 800 of Flowmeters

Two key parameters of the solar collector were analyzed: the thermal efficiency of the collector and the temperature rise of the fluid. The thermal efficiency, denoted as ɳ, was determined using the stationary thermal efficiency method outlined in the UNE-EN 12,975–2:2006 standard^26^, as well as in earlier literature^2,4,27,28^. The value of the thermal efficiency is calculated using Eq. (2):

2 $$\eta = \dot{m} \cdot c_{p} \left( {T_{o} - T_{i} } \right)/AG$$

where $\dot{m}$ is the mass flow rate, *T*_*o*_ and *T*_*i*_ are the outlet and inlet temperatures (K), respectively, A is the plate absorber area of the collector (m^2^), and G is the solar irradiance (W m^−2^). The efficiency, η, has been represented as a function of the reduced temperature T*, given by Eq. (3).

3 $$T^{{*}} = T_{{f}} - T_{a} /G$$

where *T*_*f*_ is, according to UNE-EN 12,975^26^, the mean fluid temperature within the collector and *T*_*a*_ is the ambient temperature.

The heat absorbed by the fluid or useful heat ${q}_{u}$ is given by Eq. (4)

4 $${q}_{u}={\dot{m\cdot }c}_{p}\cdot \left({T}_{o}-{T}_{i}\right)={\dot{V\cdot }\delta \cdot c}_{p}\cdot \Delta T$$

where $\dot{V}$ is the volumetric flow rate through the collector (m^3 ^s^−1^), calculated using the specific heat of the fluid (c_p_) and its density (ρ) at an average temperature of 40 °C. For water, the specific heat value is approximately 4179 J·kg^−1 ^K^−1^, and the density is about 999 kg·m^−3^.

## Results and discussion

### Graphene oxide characterization

#### Surface morphology

**Figure 2:**
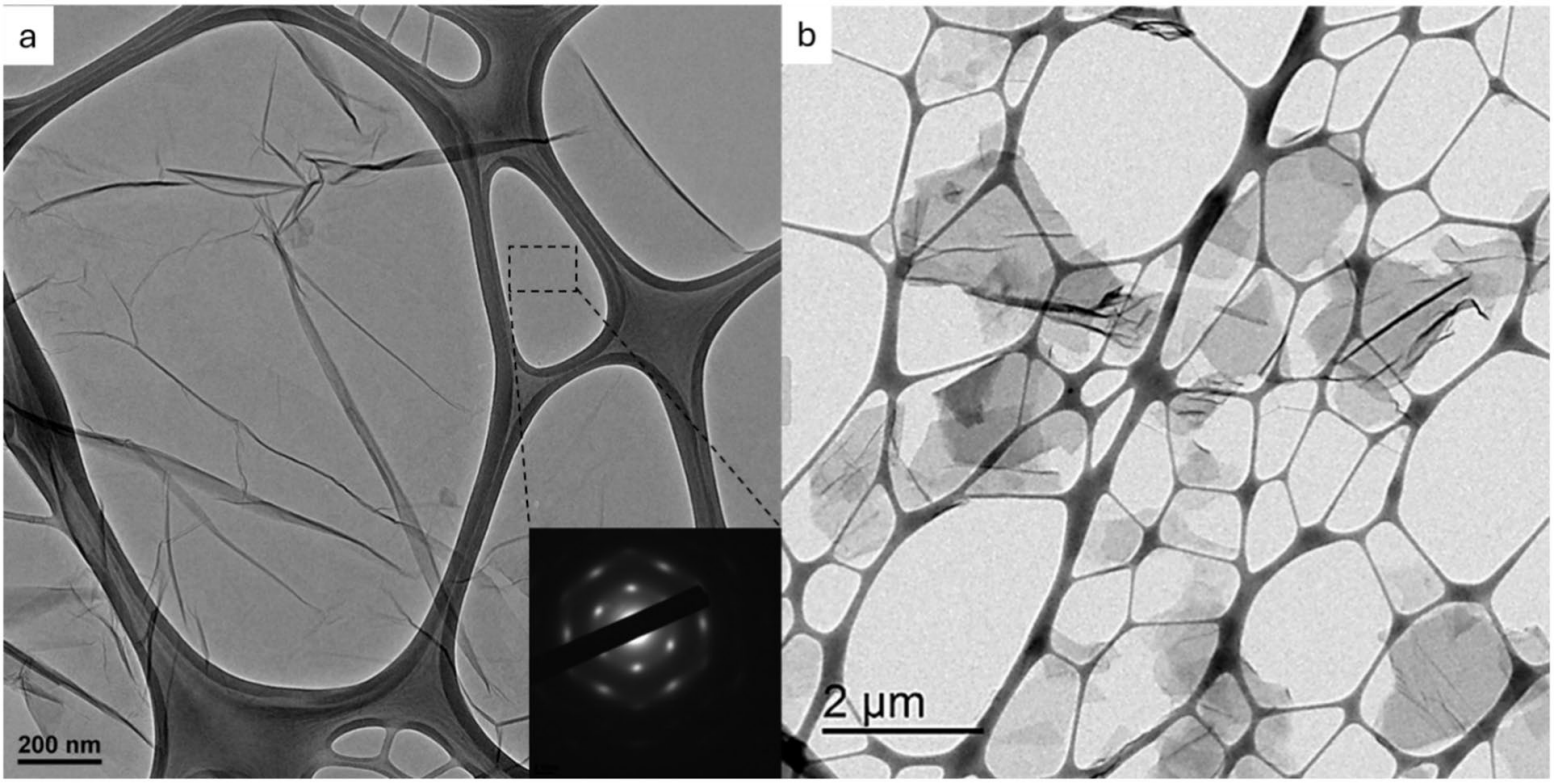
TEM micrographs of graphene oxide (GO).

Figure 2 shows the TEM images of the GO sheets used in this study, which present high crystallinity. The oxidation treatment facilitates the separation of layers from the parent graphite. GO sheets present typical graphene corrugations^29^ wherein the average size of the GO platelets is ca. 1–3 μm. The yield in monolayer sheets is above 50%, which was ascertained by TEM statistical analysis^30^. Figure 2 shows an overview of GO sheets: Fig. 2a shows an individual sheet, including in the insert the electron diffraction pattern which corresponds to a single layer, while Fig. 2b shows an overview of the sheet sizes of a set of platelets in a lower magnification image. Additionally, Figure S1 shows the elemental composition mapping carried out by STEM coupled with super X-EDS analytical system, where the distribution of oxygen is homogeneous along the sheet dimensions.

#### Surface chemistry

**Figure 3:**
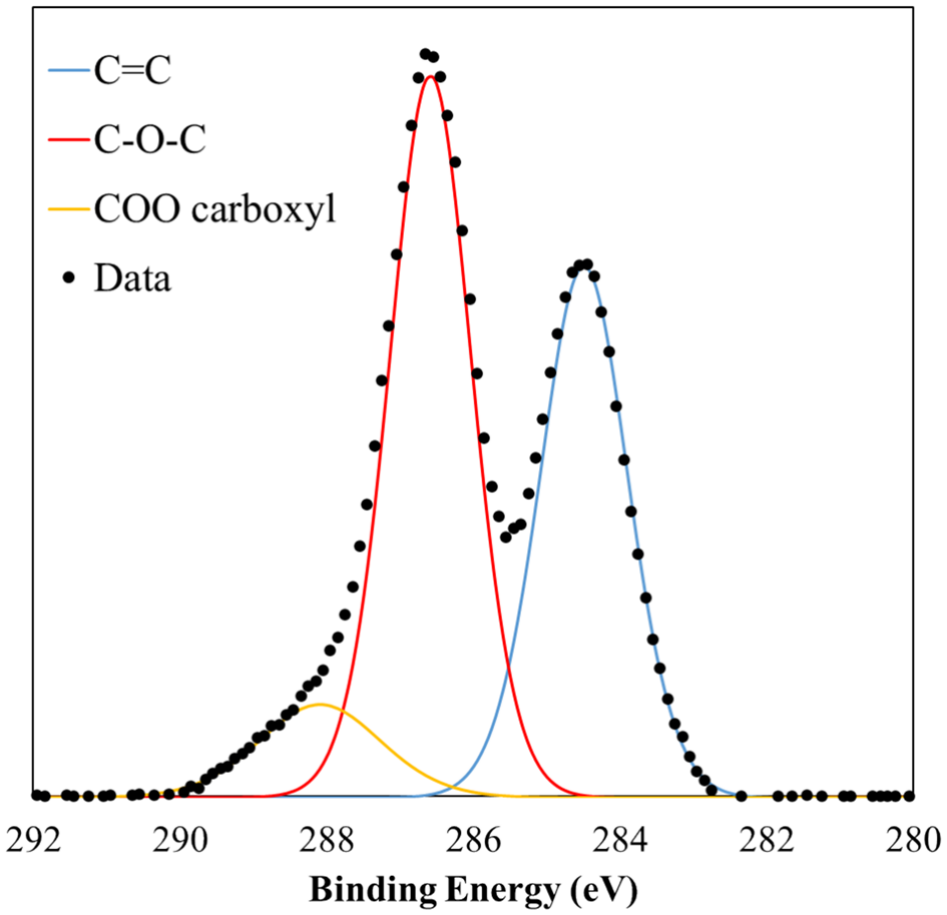
XPS C1s spectra of the GO and their deconvolution (C=C groups: blue line, C–O–C: red line and COO groups: orange line).

Figure 3 presents the results of C1s XPS spectrum corresponding to the GO sample. The displayed pattern is typical of a conventional graphene oxide, as the literature shows^31–33^. In this way, spectrum reveals a clear sp2C-sp2C peak at 284.5 eV and another minor peak at 288.5 eV assigned to either lactone or carboxylic groups^33,34^. On the other hand, the largest contribution corresponds to the range between 285 and 287.6 eV, centered at 286.5 eV, which involves binding energies attributed to hydroxyl groups (285.9 eV), ether epoxy groups (286.5 eV) and both lactols and carbonyl groups (ca. 287.6 eV)^31,32^. Therefore, the presence of ether or epoxy groups predominates in the GO sample.

#### Thermogravimetric analysis

**Figure 4:**
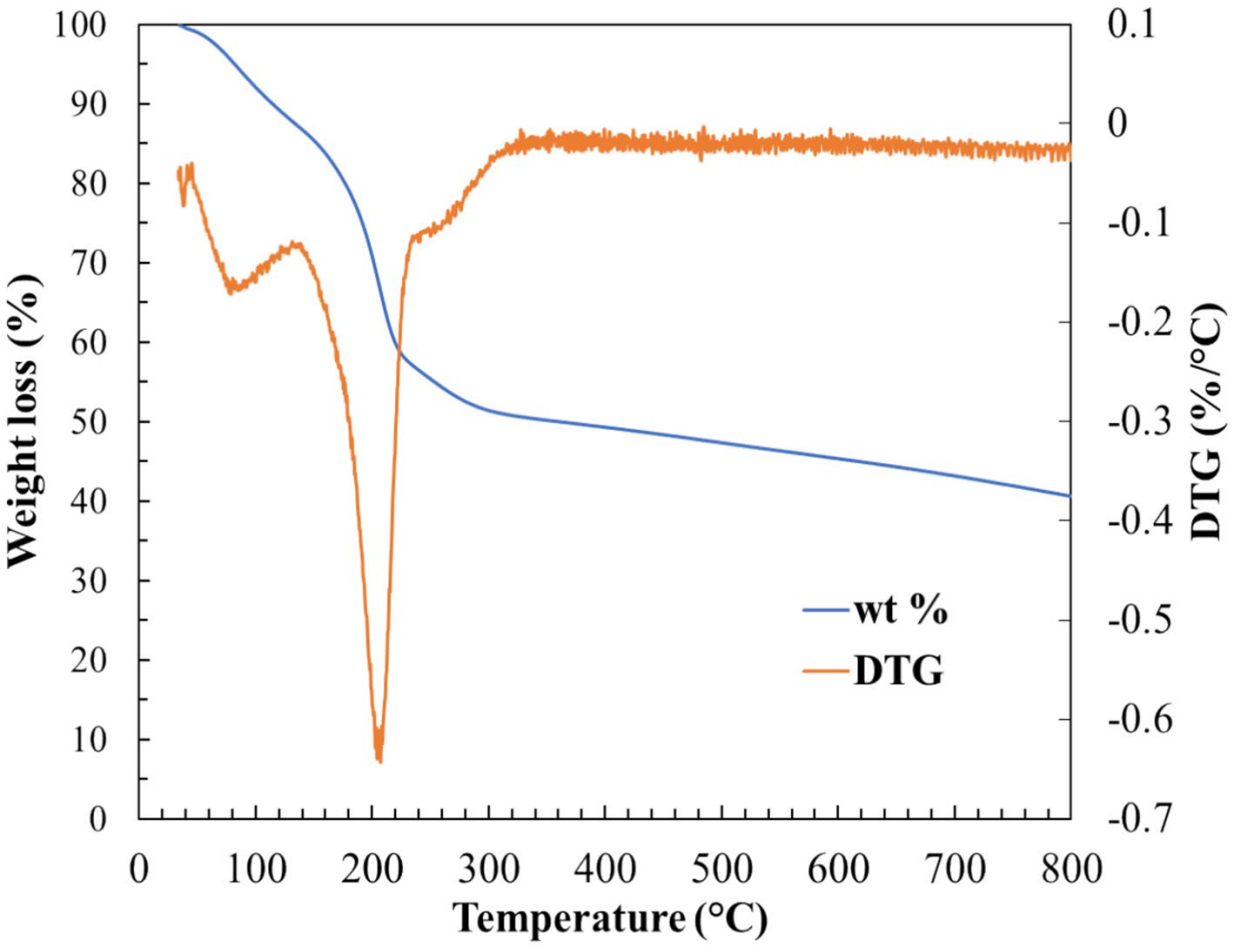
Thermogravimetric analysis in inert atmosphere of GO.

The TG analysis of GO is plotted in Fig. 4 showing three weight loss steps consistent with literature^35,36^. The initial step ranging from room temperature to 140 °C is attributed to physiosorbed H_2_O, which corresponds to a weight loss of about 13%. The second weight loss, around 27%, is observed between 140 and 220 °C and corresponds to the decomposition of oxygen groups in the form of H_2_O, CO and CO_2_^30,37^. The weight loss observed between 220 and 300 °C, roughly 8%, may be attributed to the decomposition of organosulfates^38^. Finally, a gradual smooth weight loss is observed, attributable to oxygen groups or even carbon defects of different thermal stability^37^.

#### X-ray diffraction analysis of the GO

**Figure 5:**
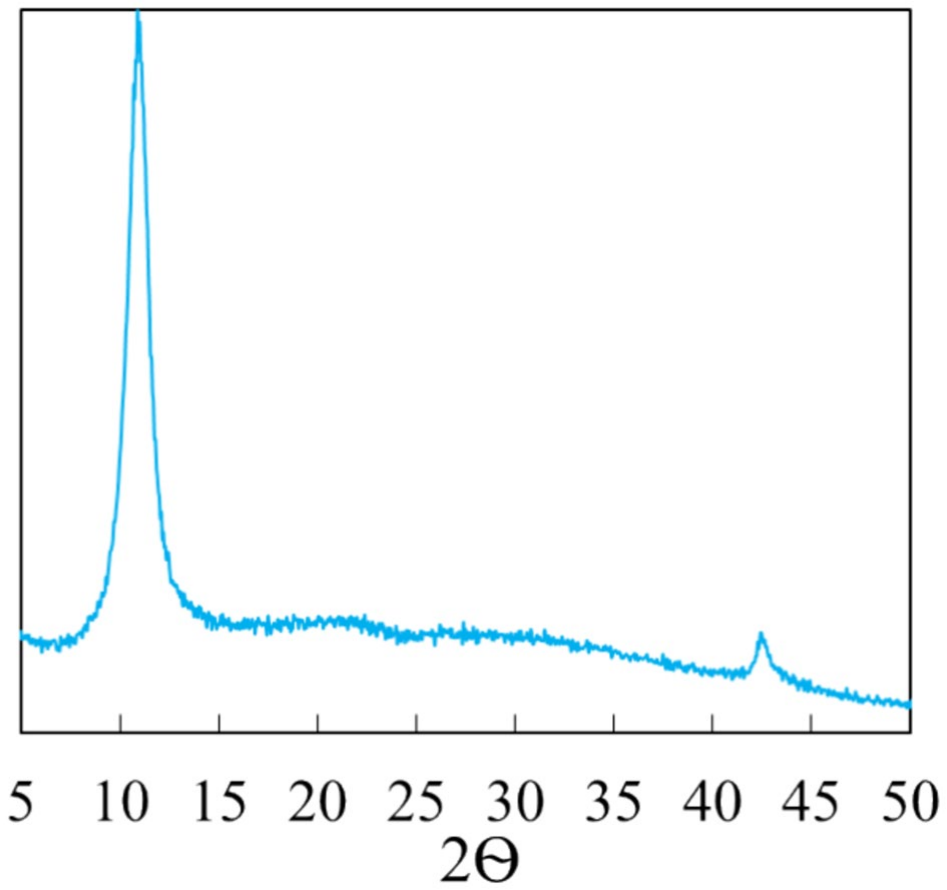
XRD patterns of graphene oxide (GO).

Figure 5 displays the X-ray diffraction (XRD) spectrum of GO. The XRD pattern shows the characteristic (002) peak of graphene oxide at 10.9°, which corresponds to a specific d-spacing of 8.1 Å between the remaining stacked layers, as previously reported by Marcano et al.^39,40^. Additionally, the XRD plot exhibits a second peak at 42.8° corresponding to 101 plane, which regularly is not so clearly observed, indicative of high crystalline sheets^39,40^.

#### ATR-FTIR analysis of the GO

**Figure 6:**
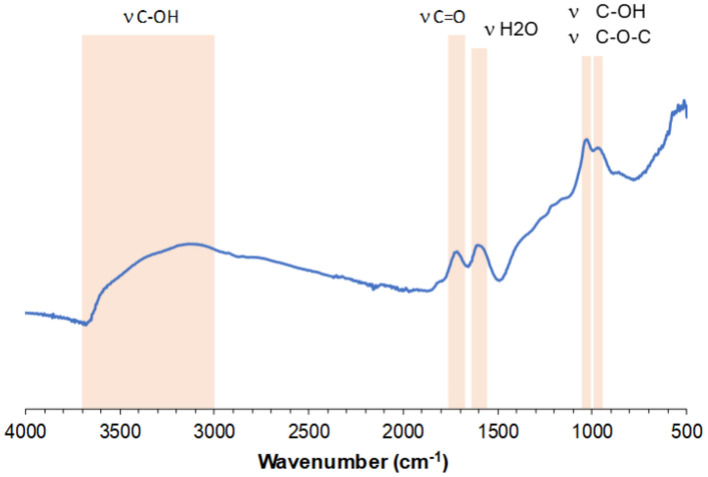
ATR-FTIR spectra of GO.

The ATR-FTIR spectrum of GO (Fig. 6) reveals several important features. Firstly, the broad band at 3000–3700 cm^−1^ can be assigned to –OH bonds, with possible contributions of water and hydroxyl groups^41^. The peak observed at 1731 cm^−1^ can be attributed to the presence of any C=O groups (either carboxyl, lactones or carbonyls^42,43^). Furthermore, band at 1620 cm^−1^, evidences adsorbed water, overlapping possible presence of localized C=C bonds in the GO structure which would have a weaker signal at 1580 cm^−1^^42,43^. The double band at 975–1050 cm^−1^ can be due to the presence of epoxides and hydroxyls, respectively, major groups in GO. Shoulder peak at 1225 cm^−1^, is attributed to O-S stretching vibrations due to the presence of organosulfate groups^30,44^, corroborating the interpretation of TG-DTG.

### Thermophysical properties of the INFs

#### Dynamic light scattering

**Figure 7:**
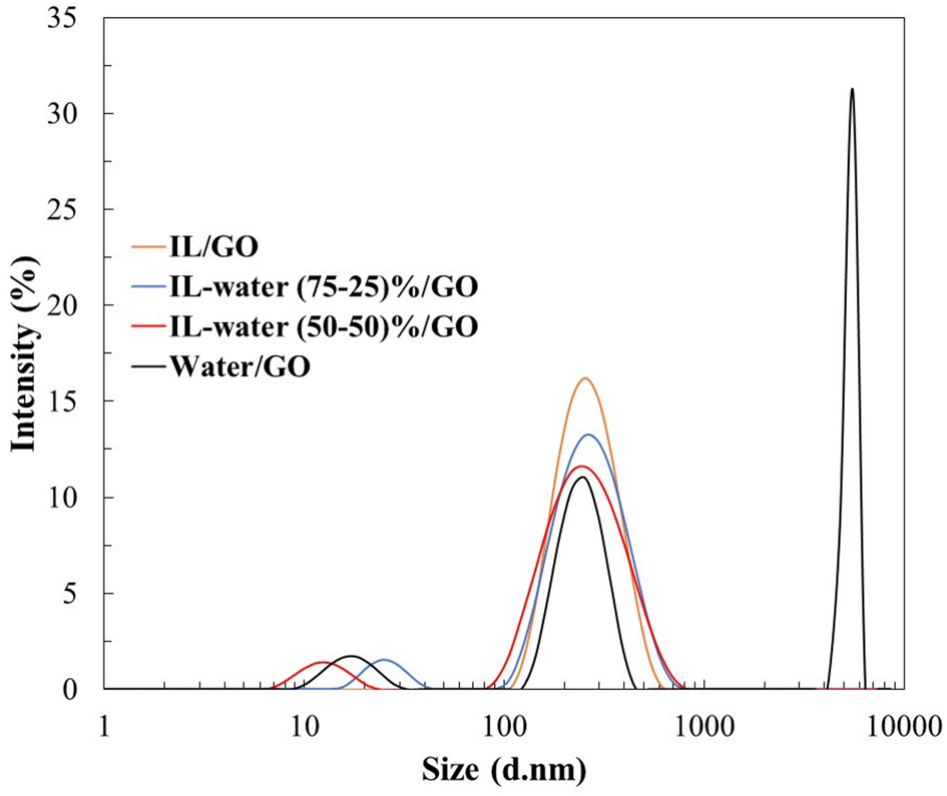
Particle size distribution of the INFs.

The synthesized INFs were characterised by DLS to determine their hydrodynamic diameter (Z-Average), polydispersity (PDI) and the respective peaks of the size distribution (Table 6 and Fig. 7).

**Table 6:** Particle size distribution of INFs prepared.

INFs	Z-Average (nm)	PDI	Peak 1 (d.nm)	Peak 2 (d.nm)	Peak 3 (d.nm)
IL/GO	180.0 ± 30.03	0.495 ± 0.095	297.0 ± 94.15	–	–
IL-water (75–25) %/GO	150.0 ± 4.008	0.583 ± 0.029	307.1 ± 145.4	20.33 ± 5.065	–
IL-water (50–50) %/GO	166.0 ± 11.42	0.541 ± 0.015	287.0 ± 110.9	25.67 ± 5.292	–
Water/GO	2107 ± 703	1.000 ± 0000	248.6 ± 61.42	5395 ± 312.7	17.48 ± 4.286

As can be seen in Table 6, the presence of water led to less homogeneous size distributions, resulting in significantly larger size, which can be attributed to the poor dispersion of GO in water.

#### Stability analysis of INFs by means of Turbiscan

**Figure 8:**
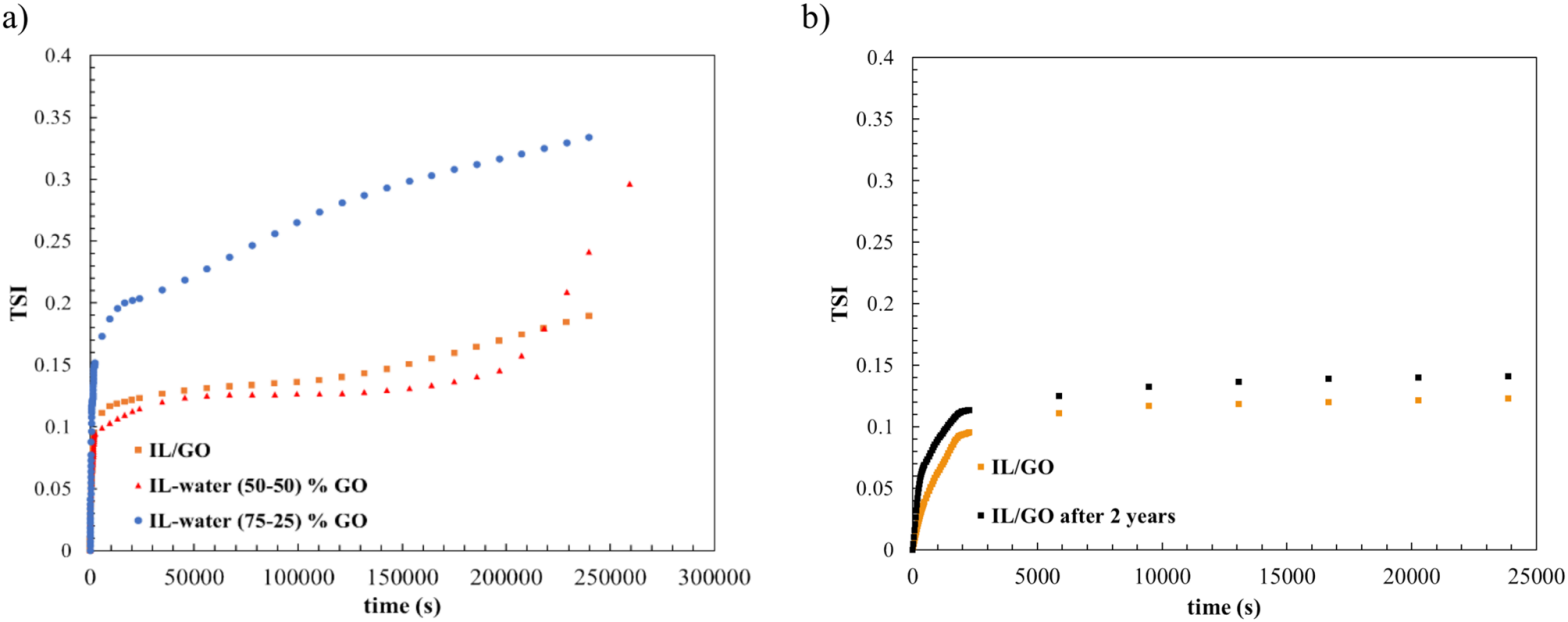
Stability index over time of: (**a**) Different INFs obtained, (**b**) IL/GO at the time of preparation and after two years.

The stability of the samples was assessed using Turbiscan^®^LAB™ for 48 h to determine their TSI. Figure 8 depicts the TSI trend as a function of time for each sample at 25 °C. As observed, all the INFs exhibit a TSI trend of around 0.2 on the first day, followed by a slight increase in TSI values on the second day, indicating the high stability of these samples. Moreover, certain authors have reported that a lower TSI value signifies high stability of the sample^45,46^. This suggests that when the TSI value is closer to zero or at its minimum, the nanoparticle suspension is less susceptible to settling or aggregation, and it is considered more stable over time. Conversely, higher TSI values suggest that the sample is less stable, indicating potential concerns related to particle aggregation or sedimentation.

On the other hand, and in order to confirm the stability over long term, the sample was subjected to Turbiscan analysis two years after its initial preparation. The results indicated high stability (Fig. 8B), as confirmed by a TSI value of approximately 0.14, the same value as before.

#### Density of the INFs

**Figure 9:**
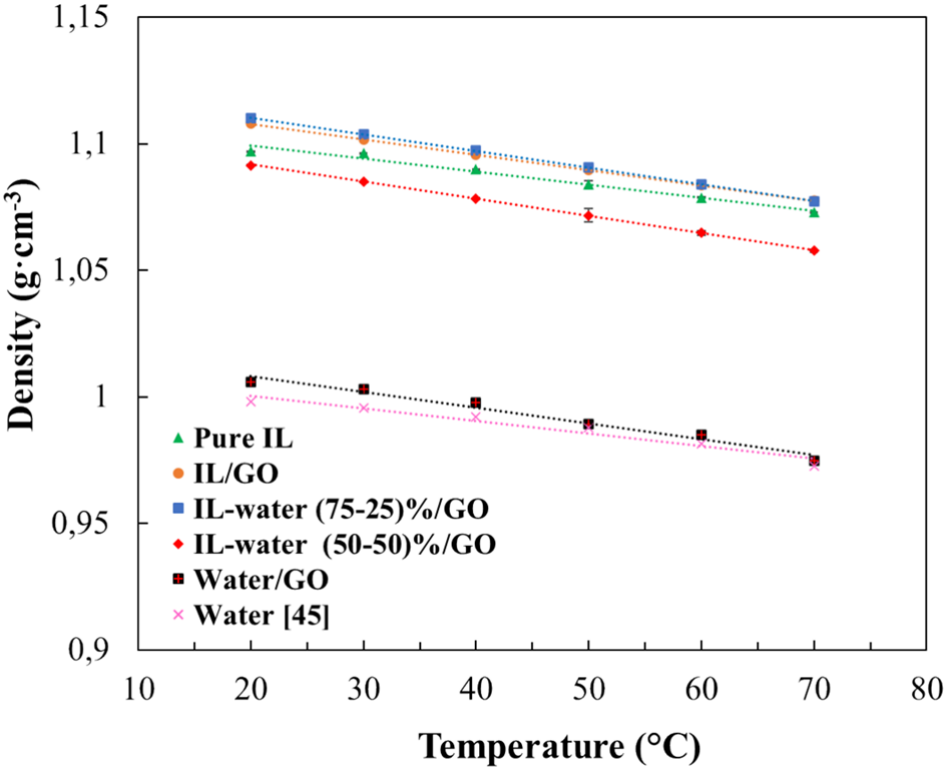
Density of pure IL and INFs as function of temperature.

Density is a fundamental property for understanding the behavior of heat transfer fluids, since denser fluids exhibit higher thermal conductivity^47^. The density results of the INFs as a function of temperature are displayed in Fig. 9. As expected, the density values decrease with the increase of the temperature. It can be seen that the density of all the prepared INFs is greater than that of the nanofluid with water alone as the base fluid. This is due to the higher density of pure IL compared with water. In addition, comparing the samples with only water or only IL with those containing GO, it can be concluded that the presence of GO increases the density values.

The experimental density values of pure IL and INF (supplementary information Table S1) were correlated through linear regression with temperature, and the fitting parameters and regression coefficients are presented in supplementary information (Table S6).

#### Viscosity of the ionanofluids

**Figure 10:**
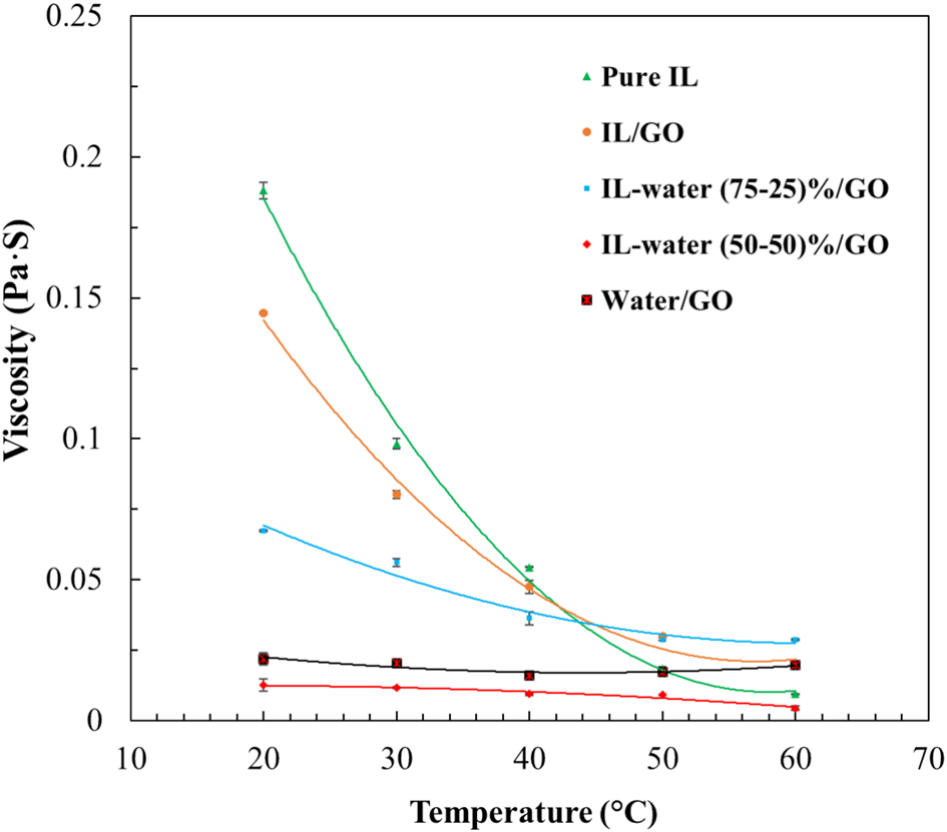
Viscosity of IL and different INFs as function of temperature.

Viscosity is a critical property of heat transfer fluid, because, a high viscosity implies more energy consumption, resulting in pressure drop in heat transfer equipments^47^. Figure 10 shows the viscosity of the pure IL and the INFs at temperature range between 20 and 70 °C. It is clear that the pure IL displayed the highest viscosity between all the base fluids investigated and that the viscosity decreases with the addition of water to the IL. In addition, as expected, the viscosity decreases with rising temperature in all cases. The results can be explained by the increase in the random velocity of the nanoparticles, leading to a reduction in the intermolecular forces between the base fluid and the nanoparticle surface, resulting in a decrease in the viscosity of the ionanofluid^47^. Many authors have also illustrated the decrease of the viscosity of nanofluids with increasing temperature^48–50^, similar to the behavior observed in density.

The experimental viscosity values of pure IL and INF (supplementary information Table S2) were correlated using a second-degree polynomial with temperature and the fitting parameters and *r*-squared are shown in Table S7 of supplementary information.

To determine if a fluid is Newtonian using data obtained from a rotational viscometer, the Newton Law for fluids can be checked. As it is known, a fluid is considered Newtonian if the relationship between shear stress (τ) and shear rate (γ) is linear and constant, which can be expressed with Eq. (5):

5 $$\uptau =\upeta \cdot\upgamma$$

where η is the dynamic viscosity of the fluid, which is constant for a Newtonian fluid at specific temperature and pressure conditions.

Figure S3 shows the relationship between the shear stress and shear rates at different temperatures for the IL and IL/GO samples. As can been seen, there is a linear relationship between both parameters for all the temperatures and the dynamic viscosity of the fluid is constant for the different shear rates. This clearly indicates the Newtonian behavior of the samples.

#### Refractive index

**Figure 11:**
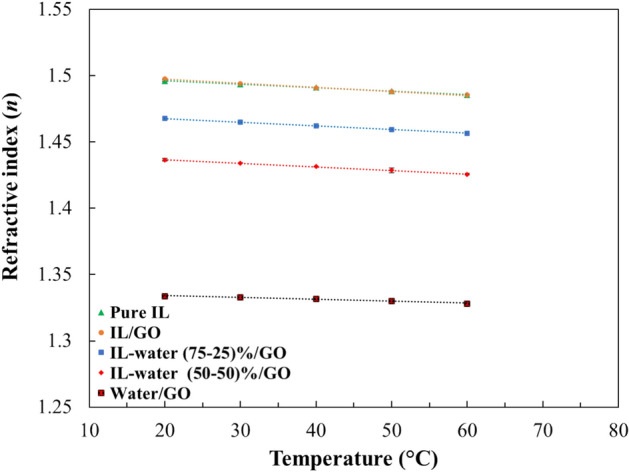
Refractive index of the pure IL and INFs as function of temperature.

The refractive index (*n*) is related to the speed at which light rays propagate through a specific medium. It can be expressed as a ratio between the speed of light in the given medium and in vacuum. Figure 11 summarizes the results obtained. The refractive index of pure IL was higher than the INFs prepared from IL-water mixture as base fluid, and it was observed that by the value decreases with an increase in temperature. Moreover, the refractive index of the sample water/GO is quite lower than others, which can be attributed to the base fluid being water, as demonstrated in previous research^51^.

The experimental refractive index values of the pure IL and INF (supplementary information Table S3) were correlated using linear regression with temperature, and the resulting fitting parameters and regression coefficients are presented in Table S6 of supplementary information.

#### Thermal conductivity

**Figure 12:**
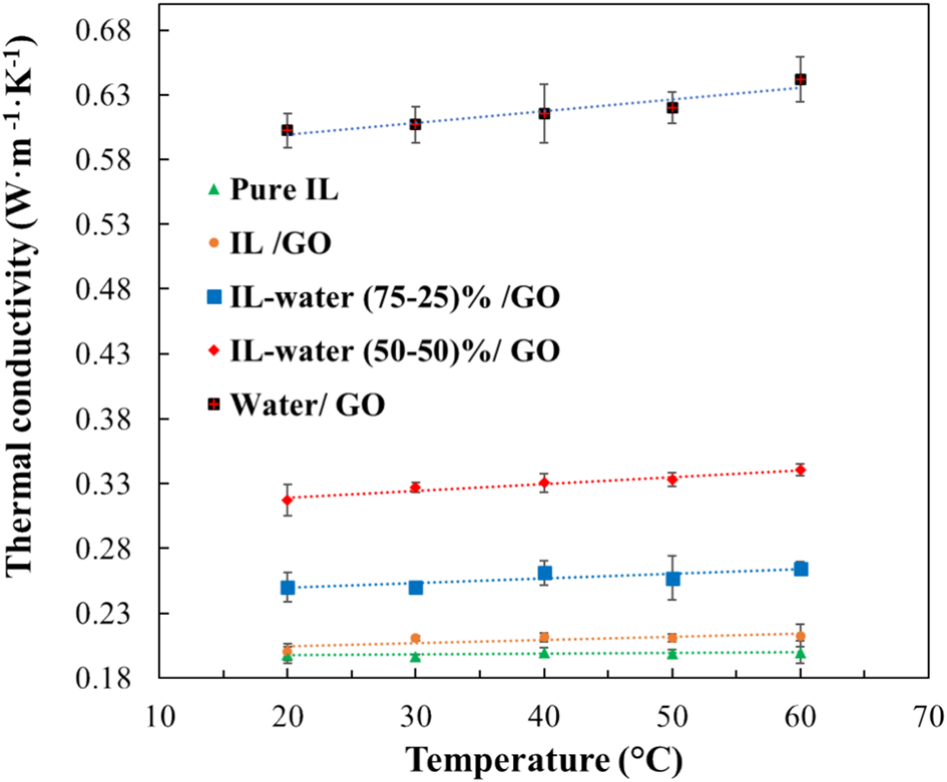
Thermal conductivity of the pure IL and INFs as a function of temperature.

Thermal conductivity of the prepared INFs was evaluated at temperatures ranging from 20 to 60 °C. As depicted in Fig. 12, thermal conductivity of the pure IL practically remains constant. However, the addition of GO leads to an improvement in thermal conductivity, increasing from 0.1974 W m^−1 ^K^−1^ for the pure IL to 0.2106 W m^−1 ^K^−1^ for the IL/GO sample. In addition, in the samples with GO, the thermal conductivity experiences a slight increase with temperature.

Moreover, the INFs prepared from IL-water mixtures as base fluid showed a significative increase in thermal conductivity, being almost triple compared with IL/GO INF. This value increased with the increase of water fraction. This result is in concordance with literature, where the effect of water fraction on the thermophysical properties of [Emim]Ac was studied^52^.

The experimental thermal conductivity values of the pure IL and INF, shown in Table S4 of the supplementary information, were correlated using linear regression with temperature. The fitting parameters and regression coefficients are provided in the supplementary information (see Table S8). The uncertainty analysis of the thermal conductivity measurements and the combined standard uncertainty results for each temperature are also developed in the supplementary information (see Table S9).

#### Specific heat capacity

**Figure 13:**
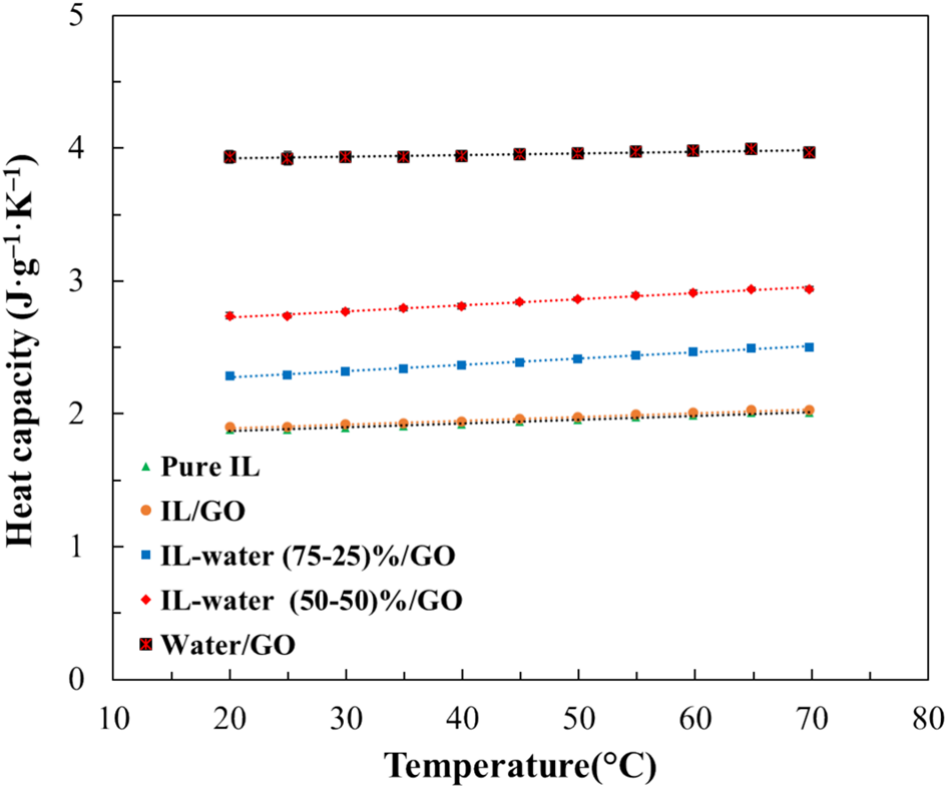
Specific heat capacity of the pure IL and INFs at different temperatures.

Heat transfer systems are based on the thermal properties of the fluids. In this context, a high heat capacity is advantageous for enhancing thermal storage density. Figure 13 illustrates the heat capacities of the pure IL and the prepared INFs, including a nanofluid of water/GO, as a function of temperature. It was observed that the pure IL has similar values of the specific heat capacity as the IL/GO, indicating that the addition of GO has not a significant effect on its heat capacity. However, the addition of GO to the water-IL mixtures showed a significative improvement in heat capacity, increasing with temperature, which is associated to the high specific capacity of the water. Nevertheless, some authors have reported that the dispersion of graphene, carbon black and Al_2_O_3_ in an IL decreases the specific heat capacity of the INF^49,53^. In addition, Zhang et al^52^ reported that heat capacity of binary solutions decreases with the increase of IL fraction, attributable to the lower specific heat capacity of [Emim] Ac.

The experimental specific heat capacity values (supplementary information Table S5) of the pure IL and INFs were correlated using linear regression with temperature, and the fitting parameters and regression coefficients are detailed in supplementary information Table S8.

#### Heat transfer coefficient

The heat transfer coefficients were evaluated in order to determine the efficiency of the INFs in the solar thermal pilot plant. The ratio of the heat transfer coefficients was calculated using the Dittus–Boelter correlation^54^, a formula previously used by other researchers to analyze the efficiency of concentrating solar power plat^55,56^, which is given by Eq. (6):

6 $${h}_{nf}/{h}_{bf}={({\rho }_{nf}/{\rho }_{bf} )}^{0.8} {\cdot ({k}_{nf}/{K}_{bf} )}^{0.6} \cdot ({{{C}_{p}}_{nf}/{{C}_{p}}_{bf} )}^{0.4} {({\mu }_{nf}/{\mu }_{bf})}^{-0.4}$$

where *h* is the heat transfer coefficient, *ρ* is the density, *k* the thermal conductivity, *C*_*p*_ the specific heat capacity and *μ* is the dynamic viscosity. All these parameters have been previously determined. *nf* and *bf* are associated with the INF and the base fluid, respectively. According to Dittus-Boelter, if ${h}_{nf}/{h}_{bf}$ obtained is $>1$, the efficiency of the system is enhancing^54^.

**Figure 14:**
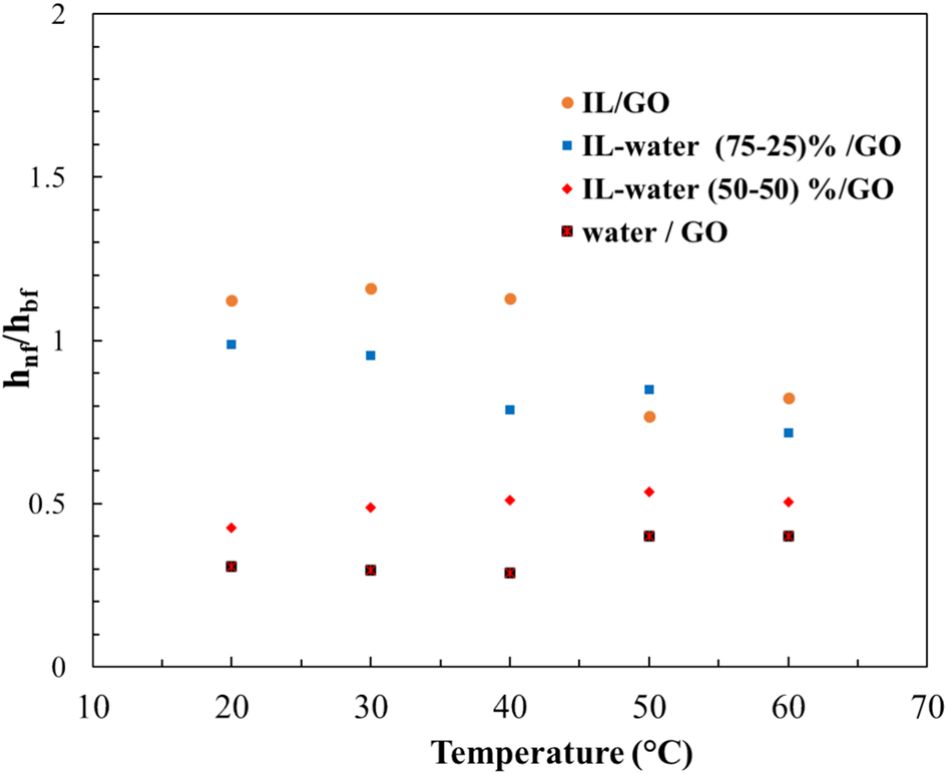
Heat transfer coefficient ratio of different INFs as a function of temperature.

Figure 14 presents the heat transfer coefficient ratio as a function of temperature. As can be seen, the ${h}_{nf}/{h}_{bf}$ ratio decreases when the water fraction in the base fluid increases. The highest ratio was obtained for the IL/GO INF, showing ${h}_{nf}/{h}_{bf}$ $>1$, indicative of a favorable improvement in the efficiency of the solar collector plat.

#### Thermal stability of INFs

**Figure 15:**
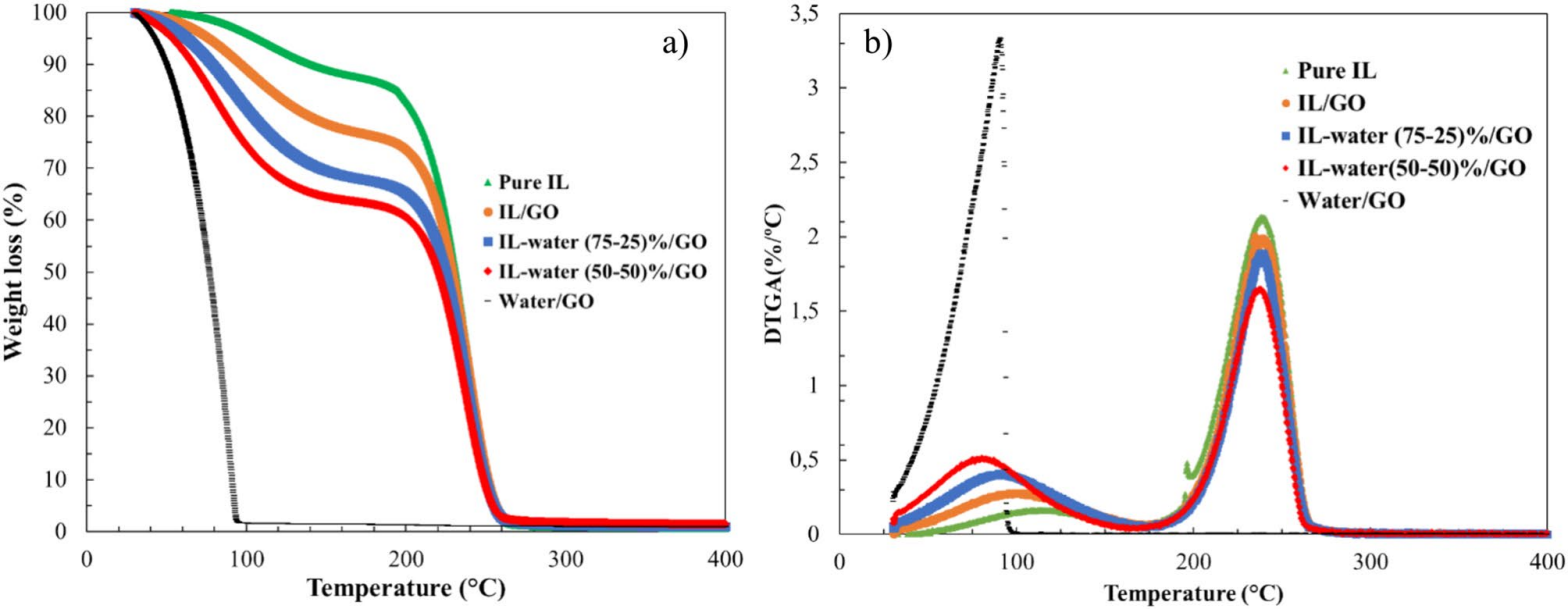
Thermogravimetric analysis in inert atmosphere of pure IL and INFs obtained; (**a**) TG and (**b**) DTGA.

One of the main causes of heat transfer and lubrication fluid failure in thermal systems is thermal degradation caused by high temperature conditions. Thermal analysis was conducted to ascertain the thermal breakdown of the INFs, in order to circumvent this. Accordingly, the samples were heated from room temperature to 800 °C at a rate of 10 °C/min under an inert atmosphere of nitrogen. Figure 15 displays the thermal degradation of the four INFs samples and pure IL. With continual weight loss, the initial decomposition temperature increased with an increase in the water fraction. The water/GO nanofluid showed the highest thermal degradation and presented a total degradation of about 100 °C, likely attributed to the boiling point of water, and the effect of GO decomposition cannot be seen according to GO concentration is just 1 wt. %. According to the results obtained, the addition of GO nanoparticles did not affect the thermal stability of the INFs, as the pure IL displayed the same trend as the INFs. [Emim] Ac is known to be highly hygroscopic, as it can be observed from the TG pattern of only IL, which shows the known effect of non-ideal behavior of [Emim] Ac-water due to strong H-bond network^57,58^. When 1% GO is incorporated, more water is uptaken by IL. Regarding the thermal stability, it was observed that all INFs suspensions were completely degraded at 250 °C. The results suggest that the INFs obtained are highly stable and present more or less the same stability as pure IL, excepted the nanofluid prepared using water as base fluid, concluding that the change of the base fluid from water to ionic liquid led to the development of a fluid with high thermal stability.

### Solar collector tests

After extensive characterization of the different prepared INFs, the IL/GO was selected to be tested in FPSC, alongside pure IL and water, due to their high stability previously demonstrated by DLS and Turbiscan (TSI value) and its high heat transfer coefficient ratio compared with the other INFs. Table 7 shows the average values obtained in the tests carried out with the different fluids. As can be seen, the IL/GO INF yielded the best results in terms of efficiency and thermal gradient.

**Table 7:** Coefficients and efficiency of the pilot solar collector using water, pure IL, and IL/GO INF.

	Flow rate (L/h)	∆T (K)	Efficiency (%)
Water	12.7	3.7	32.6
Pure IL	11.5	7.9	43.4
IL/GO	11.0	9.5	52.1

The thermal efficiency of the collector using IL/GO was found to be 52.1%, which is 16.7% higher than when using pure IL and 37.4% higher than in the case of water. This result might be considered surprising, given that water is generally considered to have superior thermal properties, such as higher specific heat and thermal conductivity compared to pure ionic liquids and IL/GO.

**Figure 16:**
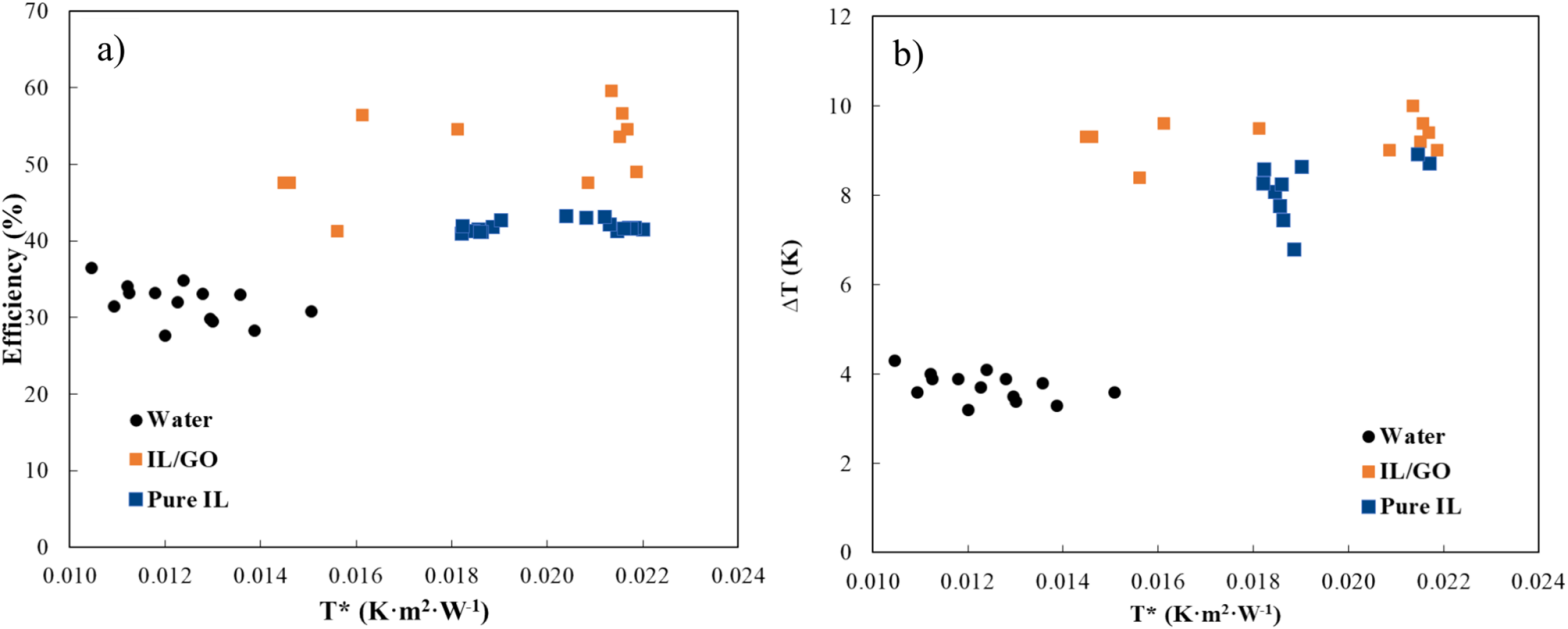
Thermal efficiency (**a**) and temperature rise through the collector (**b**) of water, pure IL and IL/GO represented against. reduced temperature T* (K·m^2^·W^−1^).

Figure 16a depicts the thermal efficiency of the collector calculated by Eq. (2), which is the parameter commonly used in collectors to estimate their performance, operating with water, pure IL and IL/GO as a function of the reduced temperature T* (K·m^2^·W^−1^) calculated by Eq. (3).

Thermal efficiency is indeed an important parameter for assessing solar collector performance. However, the increase in fluid temperature within the collector is also of significant importance as it expands the range of applications and enhances the energy quality in terms of exergy. Comparing pure IL to water, the temperature gradient inside the collector was found to be 2.1 times higher for pure IL. Moreover, with IL/GO, the temperature gradient increased even further, reaching 2.5 times that of water and 1.2 times that of pure IL. This result is expected due to the lower specific heat and density of IL/GO, which are of the same order as the INF compared to water. The higher temperature gradient signifies a greater increase in fluid temperature, which can be advantageous for various applications and indicates higher energy quality and exergy. Figure 16b represents the temperature increase through the collector of water and graphene ionanofluid as function of the reduced temperature T*.

One drawback of water as a thermal fluid is its relatively high vapor pressure. Practical applications involving liquid water near 100 ºC can be risky due to the potential for steam production. This concern is almost nonexistent with ionic liquids or their derived INFs, giving their negligible vapor pressure. Consequently, using IL/GO in a solar collector provides safer operation by eliminating the risk associated with reaching temperatures close to the boiling point of water. The results obtained indicate that the use of INFs as the heat carrier in flat plate solar collectors (FPSCs) does not result in lower thermal efficiency compared to using water. In fact, the temperature rise achieved with INFs is significantly higher. FPSC technology is widely employed for various purposes such as domestic hot water (DHW) production, household heating, and swimming pool water heating. Typically, conventional FPSC systems operate within the temperature range of 60–80 °C, primarily because these temperatures are suitable for DHW production, which is a common application of such systems. Additionally, there is a loss of efficiency at higher temperatures due to thermodynamic limitations^4^. Moreover, there is a safety concern when approaching temperatures close to 100 °C with water, as it can lead to steam production within the collectors. By using INFs in FPSCs, it becomes possible to achieve higher temperatures without compromising thermal efficiency. Reaching temperatures of 100 °C or even higher would expand the range of applications for FPSCs. This expansion would contribute to the utilization of renewable thermal energy, with solar energy being one of the most significant sources. The broader application of renewable thermal energy aligns with current objectives of public energy policies, which aim to promote and enhance the use of renewable energy sources^59^.

One significant finding of this work is the stability of IL/GO INFs, which is a crucial characteristic for scaling up their applicability in commercial plants. Comparatively, Chen et al.^60^ reported the remarkable stability of IL-based on 1-Hexyl-3-methyl-imidazolium tetrafluoroborate, [Hmim][BF_4_] silicon carbide INFs for 20 days without any sedimentation. In the present study, IL/GO remained stable even two years after preparation and testing on the solar rig, showing no signs of deterioration. During the tests, there were no notable events recorded related to the pump or other components of the plant. This indicates that IL/GO can be effectively used without causing any significant issues or damage to the system. However, it should be noted that GO nanoparticles in IL/GO are challenging to completely remove. This could be a potential drawback in laboratory test beds where different substances are tested, requiring extreme precautions. However, in commercial plants, where fluid change is usually not anticipated, this is unlikely to be an inconvenience.

## Conclusion

In conclusion, this study focused on the synthesis, characterization, and evaluation of new INFs containing the [Emim] Ac or [Emim] Ac/water mixtures as a dispersant and GO nanoparticles. The physicochemical properties and thermophysical characteristics of the prepared INFs were thoroughly analyzed. Furthermore, the study investigated the enhanced performance of IL/GO in solar collectors, their stability over time, and their potential for wider applications.

The results demonstrate the promising application of INFs in solar thermal energy systems. The laboratory-scale solar collector utilizing IL/GO exhibited a 37.4% higher efficiency and 2.5 times higher temperature rise within the collector compared to water. However, it is important to approach the improved performance of INFs with some caution, considering the specific circumstances that influence the behavior of ordinary FPSCs.

Furthermore, this study highlights the excellent stability of IL/GO, as it showed no signs of deterioration even one year after preparation and testing. Notable occurrences related to plant operation were not reported during or after the tests, emphasizing the relevance of stability for practical applications. These findings open up promising avenues for further research on the use of INFs to enhance and diversify the applications of FPSCs beyond domestic hot water production.

In light of these conclusions, plans for scaling tests on commercial solar devices are currently underway for the near future. This will provide valuable insights on the performance and feasibility of INFs in industrial-scale solar thermal energy systems.

### Supplementary Information

Supplementary Information.

## Data Availability

The data used to support the finding of this study are included within the article and its supplementary information files.
