# Supplementary material for: Novel application for graphene oxide-based ionanofluids in flat plate solar thermal collectors
Source: Sci Rep. 2024 Jul 30;14:17610. doi: 10.1038/s41598-024-67874-1 (PMC11289408; doi:10.1038/s41598-024-67874-1)
Supplement: Supplementary file 1 — Supplementary Information. [file 41598_2024_67874_MOESM1_ESM.docx]

**Supplementary information**

**Novel application for graphene oxide-based ionanofluids in flat plate solar thermal collectors**

I. Moulefera^1,2^, A. R. Pastor^1^ M. G. Fuster^1^, J.J. Delgado-Marín^1^*, M. G. Montalbán^1^, I. Rodríguez-Pastor^3^, A. López-Pérez^3^, I. Martin-Gullon^3^, A. P. Ramallo-González^4^, M. Alarcón^5^, G. Víllora^1^

^1^ Chemical Engineering Department, Faculty of Chemistry, Regional Campus of International Excellence “Campus Mare Nostrum”, University of Murcia, 30071 Murcia, Spain.

^2^ Department of Chemical Engineering, Faculty of Science, University of Málaga, Andalucía TECH, 29071 Málaga, Spain.

^3^ Institute of Chemical Processes Engineering, University of Alicante, Alicante, 03080, Spain.

^4^Department of Information and Communication Engineering, Universidad de Murcia, Murcia, 30100, Spain

^5^ Electromagnetism and Electronics Department, International Campus of Excellence in the European context (CEIR) Campus Mare Nostrum, University of Murcia, Spain.

Corresponding authors: [josejavier.delgado@um.es](mailto:josejavier.delgado@um.es); [gvillora@um.es](mailto:gvillora@um.es)

**Figures**


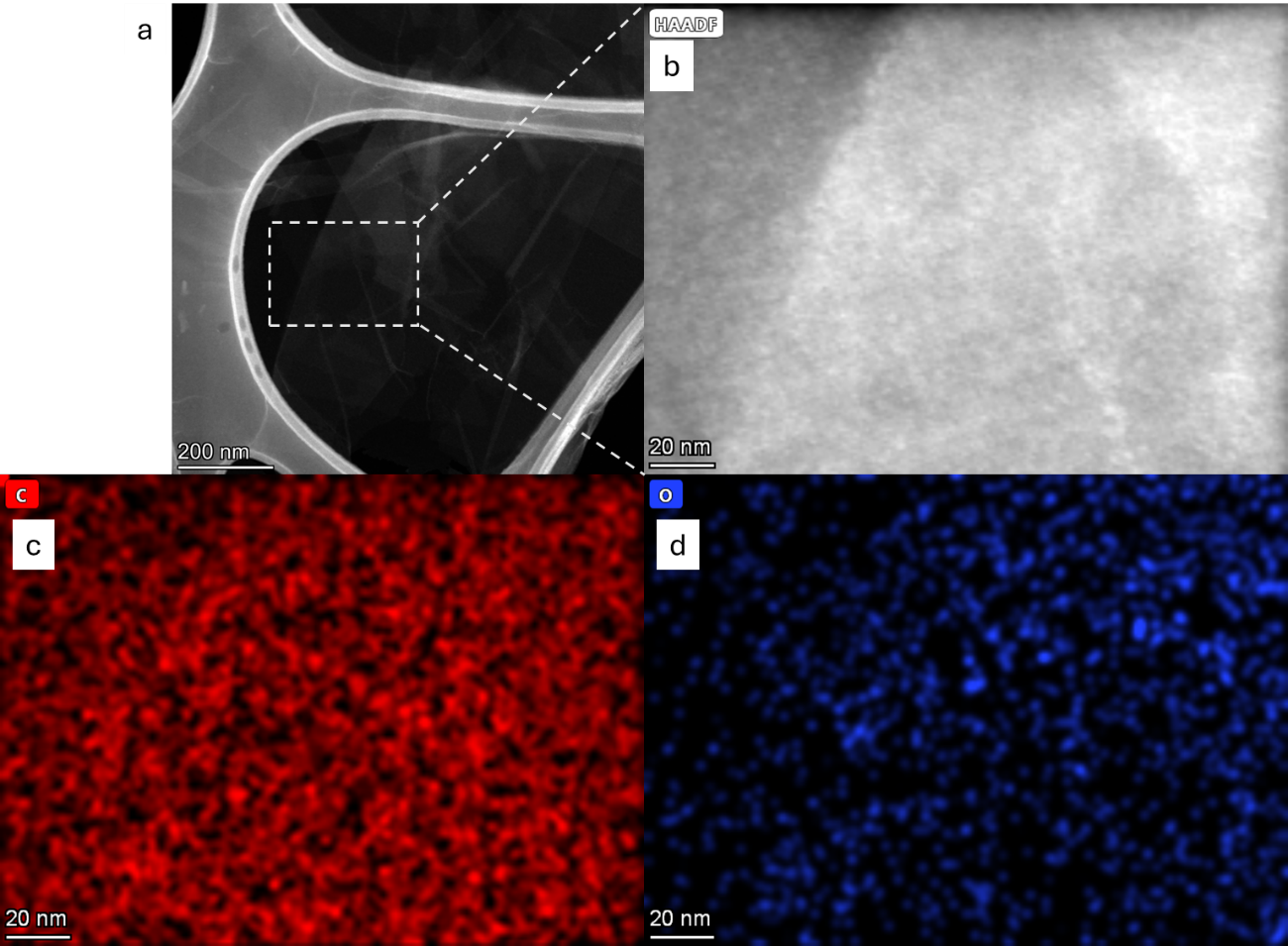


**Figure S1**. GO exploration and elemental composition mapping by STEM with super X EDS. Figure S1a shows in transmission an area where the edge of a GO sheet is clearly observed; a white rectangle marks the area where the EDS mapping is done. Figure S1b shows the mapped zone in mode high angle annular dark field (HAADF), while figures S1c and S1d shows the carbon and oxygen density. respectively. Oxygen mapping lets visualize the GO sheet, where the oxygen density is all through the sheet unless with some variation in intensity (isolated aromatic domains in a continuous sp3C zone. Oxygen concentration at the edge is similar that in basal plane.


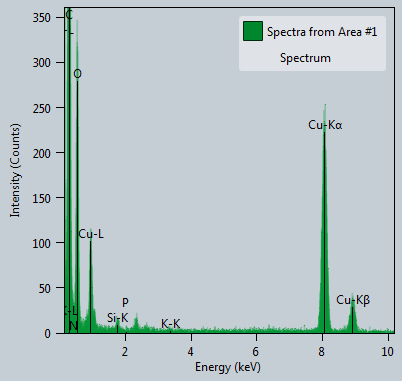


**Figure S2**. XPS spectra of the zone mapped of the graphene oxide, where C/O ratio is 3.1.

| a)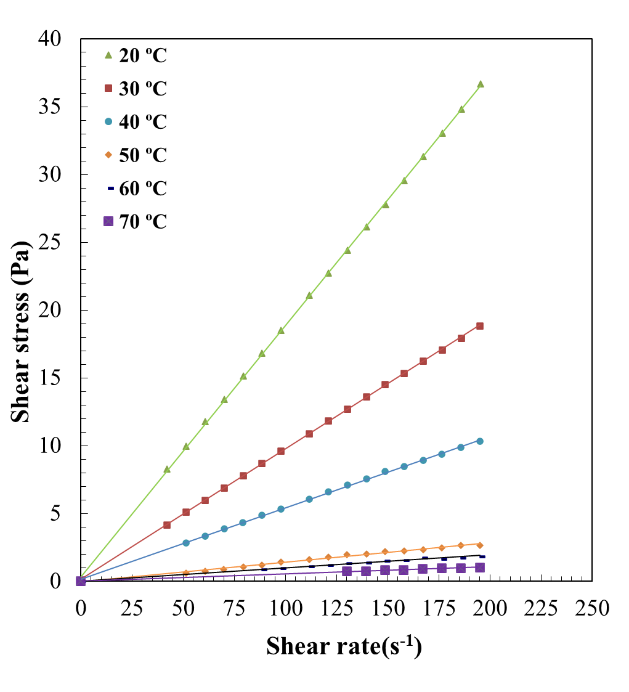 | b)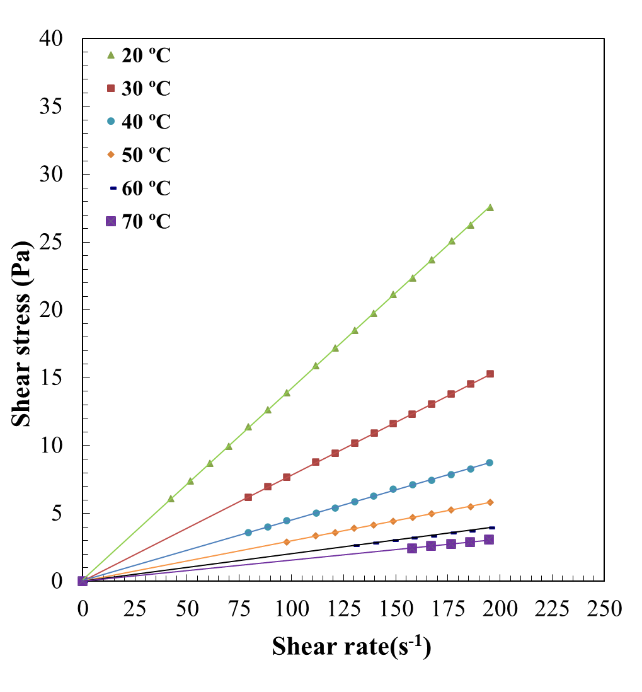 |
| --- | --- |

**Figure S3.** Shear stress *vs* shear rate for the IL (a) and IL/GO (b) fluids.

**Tables**

**Table S1.** Density of the pure IL and INFs at different temperatures.

| **T *ρ* /°C /g·Cm^-3^** | **Pure IL** | **IL/GO** | | **IL-Water (75-25)**  **%/GO** | | **IL-Water (50-50) %/G** | | **Water /GO** | |  |
| --- | --- | --- | --- | --- | --- | --- | --- | --- | --- | --- |
| 20 | 1.0851± 1·10^-5^ | | 1.1078 ± 7·10^-4^ | | 1.1102± 2·10^-5^ | | 1.0915± 6·10^-5^ | | 1.0060± 5·10^-5^ | |
| 30 | 1.0961± 9·10^-5^ | | 1.1017± 4·10^-4^ | | 1.1038± 1·10^-4^ | | 1.0851± 2·10^-5^ | | 1.0031± 7·10^-5^ | |
| 40 | 1.0901± 3·10^-5^ | | 1.0956 ± 2·10^-4^ | | 1.0973± 3·10^-4^ | | 1.0784± 5·10^-5^ | | 0.9918± 2·10^-4^ | |
| 50 | 1.0841± 1·10^-3^ | | 1.0896± 2·10^-5^ | | 1.0907± 2·10^-5^ | | 1.0716± 3·10^-3^ | | 0.9892± 6·10^-5^ | |
| 60 | 1.0786± 2·10^-4^ | | 1.0836± 2·10^-5^ | | 1.0839± 6·10^-4^ | | 1.0647± 5·10^-4^ | | 0.9852± 1·10^-5^ | |

**Table S2.** Viscosity of the pure IL and INFs at different temperatures.

| **T *µ* /°C /Pa·S** | **Pure IL** | **IL/GO** | | **IL-Water (75-25)**  **%/GO** | | **IL-Water (50-50) %/G** | | **Water /GO** | |  |
| --- | --- | --- | --- | --- | --- | --- | --- | --- | --- | --- |
| 20 | 0.1881± 3·10^-3^ | | 0.1447± 3·10^-4^ | | 0.0673± 5·10^-3^ | | 0.0126± 2·10^-4^ | | 0.0221± 2·10^-3^ | |
| 30 | 0.0983± 2·10^-3^ | | 0.0802± 2·10^-3^ | | 0.0562± 1·10^-2^ | | 0.0125± 1·10^-4^ | | 0.0204± 4·10^-4^ | |
| 40 | 0.0545± 3·10^-4^ | | 0.0475± 2·10^-3^ | | 0.0363± 7·10^-4^ | | 0.0175± 5·10^-2^ | | 0.0161± 6·10^-4^ | |
| 50 | 0.0186± 7·10^-4^ | | 0.0299± 4·10^-4^ | | 0.0288± 7·10^-4^ | | 0.0091± 9·10^-5^ | | 0.0175± 3·10^-4^ | |
| 60 | 0.0095± 6·10^-5^ | | 0.0195± 3·10^-4^ | | 0.0287± 2·10^-3^ | | 0.0044± 3·10^-5^ | | 0.0196± 8·10^-4^ | |

**Table S3.** Refractive index of the pure IL and INFs at different temperatures.

| **T *n***  **/°C** | **Pure IL** | **IL/GO** | **IL-Water (75-25)**  **%/GO** | | **IL-Water (50-50) %/GO** | | **Water /GO** |
| --- | --- | --- | --- | --- | --- | --- | --- |
| 20 | 1.4969± 5·10^-4^ | 1.4977± 6·10^-5^ | | 1.4676± 2·10^-4^ | | 1.4366± 1·10^-3^ | 1.3338± 2·10^-4^ |
| 30 | 1.4921± 4·10^-5^ | 1.4939± 1·10^-4^ | | 1.4650± 6·10^-5^ | | 1.4341± 8·10^-5^ | 1.3329± 6·10^-5^ |
| 40 | 1.4893± 1·10^-4^ | 1.4909± 6·10^-5^ | | 1.4623± 6·10^-5^ | | 1.4314± 3·10^-5^ | 1.3314± 6·10^-5^ |
| 50 | 1.4864± 5·10^-5^ | 1.4881± 1·10^-5^ | | 1.4595± 3·10^-5^ | | 1.4286± 2·10^-3^ | 1.3299± 3·10^-5^ |
| 60 | 1.4837± 1·10^-5^ | 1.4854± 5·10^-5^ | | 1.4567± 2·10^-4^ | | 1.4257± 5·10^-4^ | 1.3282± 2·10^-4^ |

**Table S4.** Thermal conductivity of Pure IL and INFs at different temperature studies.

| **T K**  **/°C /W·m^-1^·k^-1^** | **Pure IL** | **IL/GO** | **IL-water (75-25)**  **%/GO** | | | **IL-water (50-50) %/GO** | | **Water /GO** |
| --- | --- | --- | --- | --- | --- | --- | --- | --- |
| 20 | 0.1964± 5·10^-3^ | 0.2002± 6·10^-3^ | | 0.2503± 2·10^-2^ | 0.3172± 1·10^-2^ | | 0.6022± 3·10^-2^ | |
| 30 | 0.1978± 2·10^-3^ | 0.2106± 2·10^-3^ | | 0.2498± 3·10^-3^ | 0.3270± 4·10^-3^ | | 0.6069± 1·10^-2^ | |
| 40 | 0.1998± 2·10^-3^ | 0.2116± 3·10^-3^ | | 0.2611± 9·10^-3^ | 0.3306± 7·10^-3^ | | 0.6155± 2·10^-2^ | |
| 50 | 0.1985± 3·10^-3^ | 0.2112± 3·10^-3^ | | 0.2602± 2·10^-2^ | 0.3333± 5·10^-3^ | | 0.6198± 3·10^-2^ | |
| 60 | 0.1996± 8·10^-3^ | 0.2125± 9·10^-3^ | | 0.2645± 5·10^-3^ | 0.3408± 4·10^-3^ | | 0.6418± 3·10^-2^ | |

**Table S5.** Specific heat capacity of Pure IL and INFs at different temperature studies.

| **T K**  **/°C /W·m^-1^·k^-1^** | **Pure IL** | **IL/GO** | **IL-water (75-25)**  **%/GO** | | | **IL-water (50-50) %/GO** | | **Water /GO** |
| --- | --- | --- | --- | --- | --- | --- | --- | --- |
| 20 | 1.8800± 1·10^-2^ | 1.900± 3·10^-2^ | | 2.2800± 2·10^-2^ | 2.7400± 6·10^-2^ | | 3.9400± 1·10^-2^ | |
| 30 | 1.9000± 1·10^-2^ | 1.9200± 3·10^-2^ | | 2.3200± 2·10^-2^ | 2.7700± 7·10^-2^ | | 3.9400± 1·10^-2^ | |
| 40 | 1.9200± 1·10^-2^ | 1.9400± 4·10^-2^ | | 2.3600± 2·10^-2^ | 2.8100± 6·10^-2^ | | 3.9400± 1·10^-2^ | |
| 50 | 1.9600± 1·10^-2^ | 1.9700± 5·10^-2^ | | 2.4200± 2·10^-2^ | 2.8600± 6·10^-2^ | | 3.9600± 1·10^-2^ | |
| 60 | 1.9900± 1·10^-2^ | 2.0100± 5·10^-2^ | | 2.4700± 2·10^-2^ | 2.9100± 6·10^-2^ | | 3.9800± 1·10^-2^ | |
| 70 | 2.0100± 1·10^-2^ | 2.0300± 5·10^-2^ | | 2.500± 2·10^-2^ | 2.9400± 6·10^-2^ | | 3.9700± 2·10^-2^ | |

**Table S6**. Fitting parameters and Linear Regression Coefficient. r^2^. to the equations ρ=ρ_0_T+ρ_1_ for Density. ρ. and n=n_0_T+n_1_ for Refractive Index. n. of Pure IL and INFs.

| **Sample** | **10^-4^·*ρ0* /g·cm^-3^ ·°C** | ***ρ1* /g·cm^-3^** | ***r^2^*** | **10^-4^·*n0* /°C** | ***n1*** | ***r^2^*** |
| --- | --- | --- | --- | --- | --- | --- |
| Pure IL | -5 | 1.1096 | 0.9768 | 3 | 1.5015 | 0.999 |
| IL/GO | -6 | 1.1199 | 1 | 3 | 1.5034 | 0.994 |
| IL-water (75-25) %/GO | -7 | 1.1235 | 0.999 | 3 | 1.4732 | 0.999 |
| IL-water (50-50) %/GO | -7 | 1.1054 | 0.999 | 3 | 1.4423 | 0.999 |
| Water /GO | -6 | 1.2070 | 0.973 | 3 | 1.3370 | 0.989 |
| Water | -5 | 1.0105 | 0.946 | 1 | - | - |

**Table S7**. Fitting parameters and -r-square Coefficient. r^2^. to the equations µ= µ _0_T^2^+ µ _1_T_+_ µ_2_ for viscosity. µ of Pure IL and INFs.

| **Sample** | **10^-5^·** µ***0* /Pa·S·°C** | µ***1* /Pa·S·°C** | | µ***2*/Pa·S** | ***r^2^*** |
| --- | --- | --- | --- | --- | --- |
| Pure IL | 10 | -0.0141 | 0.4180 | | 0.996 |
| IL/GO | 9 | -0.0101 | 0.3081 | | 0.994 |
| IL-water (75-25) %/GO | 2 | -0.0030 | 0.1189 | | 0.970 |
| IL-water (50-50) %/GO | 1 | -0.0008 | 0.0357 | | 0.843 |
| Water /GO | 0.4 | 0.0001 | 0.0011 | | 0.944 |

**Table S8**. Fitting parameters and Linear Regression Coefficient. r^2^. to the equations k=k_0_T+k_1_ for Thermal conductivity. k. and C_p_= C_p 0_T+ C_p1_ for Specific heat capacity. C_p_. of Pure IL and INFs.

| **Sample** | **10^-4^·k*_0_***  **/W·m^-1^** | **k*_1_***  **/W·m^-1^ ·K^-1^** | | ***r^2^*** | | **10^-4^·**$\boldsymbol{C}_{\boldsymbol{p}_{\boldsymbol{1}}}$  **/J·g^-1^** | $\boldsymbol{C}_{\boldsymbol{p}_{\boldsymbol{1}}}$  **/J·g^-1^·K^-1^** | | ***r^2^*** | |
| --- | --- | --- | --- | --- | --- | --- | --- | --- | --- | --- |
| Pure IL | 0.6 | | 0.1962 | | 0.424 | 29 | 1.1813 | 0.986 | |  |
| IL/GO | 3 | | 0.1992 | | 0.617 | 29 | 11832 | 0.987 | |  |
| IL-water (75-25) %/GO | 4 | | 0.2423 | | 0.755 | 48 | 2.1776 | 0.992 | |  |
| IL-water (50-50) %/GO | 5 | | 0.3085 | | 0.952 | 45 | 2.6396 | 0.988 | |  |
| Water /GO | 9 | | 0.5805 | | 0.896 | 13 | 3.8989 | 0.815 | |  |

**Table S9.** Combined standard uncertainties of thermal conductivity measurements.

| **T**  **(°C)** | **Pure IL** | **IL/GO** | **IL-water (75-25)**  **%/GO** | **IL-water (50-50) %/GO** | **Water /GO** |
| --- | --- | --- | --- | --- | --- |
| 20 | 0.0199 | 0.0203 | 0.0276 | 0.0322 | 0.0627 |
| 30 | 0.0198 | 0.0211 | 0.0250 | 0.0328 | 0.0610 |
| 40 | 0.0200 | 0.0212 | 0.0266 | 0.0333 | 0.0626 |
| 50 | 0.0199 | 0.0212 | 0.0285 | 0.0335 | 0.0644 |
| 60 | 0.0205 | 0.0219 | 0.0266 | 0.0342 | 0.0665 |

**Uncertainty analysis for thermal conductivity measurement**

For the uncertainty analysis of the thermal conductivity measurements, three factors were considered: the thermal conductivity measurements themselves, the thermostatic bath used to achieve the desired temperatures, and the conductivity meter employed for the measurements. Conductivity measurements were performed in triplicate for each temperature. The standard uncertainty of the measurements is determined by the following equation:

$$u_{Mean}=\frac{s}{\sqrt{n}}$$

Where *s* is the standard deviation and *n* is the number of measurements (*n*=3).

The conductivity meter has an accuracy of ±10% within a range of 0.02 to 2 W/m^-1^·K^-1^. Therefore, the uncertainty of the instrument is given by the expression:

$$u_{Instr}=0.10\cdot\overline{k}$$

With $\overline{k}$ being the mean of the measurements.

The thermostatic bath used has an uncertainty of 0.5 °C. However, since the change in thermal conductivity per degree is negligible, this uncertainty is considered insignificant. Consequently, the combined standard uncertainty is determined by:

$$u_{C}=\sqrt{\left( u_{Mean}^{2}+u_{Instr}^{2} \right)}$$

After performing all calculations, the combined standard uncertainties are presented in Table S9.

**Nomenclature**

| $\dot{\boldsymbol{V}}$ | Volumetric flow rate |
| --- | --- |
| **A** | Absorber area of the collector |
| **C_p_** | Specific heat capacity |
| **G** | Solar irradiance |
| ***h*** | Heat transfer coefficient |
| **H** | Total sample height |
| **K** | Thermal conductivity |
| **ṁ** | Mass flow rate |
| ***n*** | Refractive index |
| ***q_u_*** | Useful heat |
| **scan_i_ (h_i_)** | Intensity of scanning light |
| **t** | Time |
| **T** | Temperature |
| **TSI** | Turbidity Stability Index |
| **T*** | Reduced tempreature |
| **η** | thermal efficiency |
| **μ** | Viscosity |
| **ρ** | Density |

**Acronyms**

| **[Emim] Ac** | 1-ethyl-3-methylimidazolium acetate |
| --- | --- |
| **BS** | Backscattered |
| **DLS** | Dynamic Light Scattering |
| **DSC** | Differential Scanning Calorimetry |
| **FPSC** | Plate solar thermal collector |
| **GO** | Graphene oxide |
| **IL** | Ionic liquid |
| **INFs** | Ionanofluids |
| **IR-ATR** | Infrared spectroscopy with attenuated total reflectance |
| **MLS** | Multiple Light Scattering |
| **T** | Transmitted |
| **TEM** | Transmission electron microscopy |
| **TG** | Thermogravimetric analysis |
| **XDR** | X-ray diffraction |
| **XPS** | X-ray photoelectron spectroscopy. |
